# miR-18a promotes glioblastoma development by down-regulating ALOXE3-mediated ferroptotic and anti-migration activities

**DOI:** 10.1038/s41389-021-00304-3

**Published:** 2021-02-12

**Authors:** Xinzhi Yang, Jiangang Liu, Chenci Wang, Kenneth King-yip Cheng, Hongchao Xu, Qingzhong Li, Tian Hua, Xue Jiang, Lili Sheng, Jie Mao, Zhuohao Liu

**Affiliations:** 1 Department of Neurosurgery, Shenzhen Hospital, Southern Medical University, Shenzhen, Guangdong China; 2 The Third School of Clinical Medicine, Southern Medical University, Shenzhen, Guangdong China; 3 Shenzhen Key Laboratory of Viral Oncology, The Clinical Innovation & Research Centre, Shenzhen Hospital, Southern Medical University, Shenzhen, Guangdong China; 4 School of Graduate Studies, Wannan Medical College, Wuhu, Anhui China; 5 Department of Health Technology and Informatics, The Hong Kong Polytechnic University, Hong Kong, China; 6 Joint Laboratory of Guangdong and Hong Kong on Metabolic Diseases, Guangdong Pharmaceutical University, Guangzhou, Guangdong, China; 7 Research Center for Cancer, Wannan Medical College, Wuhu, Anhui China

**Keywords:** CNS cancer, Cancer metabolism

## Abstract

The development of glioblastoma (GBM) is typically accompanied by marked changes in lipid metabolism. Oxylipins and their catalyzed enzymes lipoxygenases (LOXs) have been shown to participate in the development of cancers via multiple pathways, while the understanding of LOXs in GBM remains enigmatic. Thus, we aimed to explore the expression and functional roles of LOXs in the development of GBM. Here we showed that ALOXE3 was markedly down-regulated in human GBM. Knockdown of ALOXE3 in GBM cells fostered the orthotopic tumor growth and shortened lifespan in mice. ALOXE3 deficiency rendered GBM cells resistant to p53-SLC7A11 dependent ferroptosis, promoting GBM cell survival. Mechanistically, miR-18a directly targeted ALOXE3 and suppressed its expression and functions in GBM cells. Furthermore, ALOXE3 silencing promoted 12-hydroxyeicosatetraenoic acids (12-HETE) secretion from GBM cells, in turn, 12-HETE enhanced migration of GBM cells by activating G_s_-protein-coupled receptor (G_s_PCR)-PI3K-Akt pathway in an autocrine manner. Altogether, miR-18a/ALOXE3 axis exerts tumor promoting functions by regulating ferroptosis and migration of GBM cells. Targeting miR-18a/ALOXE3 axis may provide novel therapeutic approaches for GBM treatment.

## Introduction

Glioblastomas (GBM; WHO grade IV gliomas) are the most aggressive and lethal human brain tumors in adults, with around 15 months median survival time despite combined treatment including surgical resection, radiotherapy, and chemotherapy^1–3^. Although many signaling pathways, transcriptional factors, and miRNAs have been uncovered to play crucial roles in the development and progression of GBM, efforts to target them have yielded minimal impact on GBM patient outcomes^4–8^. Thus, further molecular insights into GBM malignant progression are urgently required for discovering novel therapeutic targets. The development of GBM is typically accompanied by marked changes in metabolism, including that of lipids^9^. Alteration in lipid metabolism can influence numerous cellular processes, including proliferation, survival, and migration^10^. A cluster of lipids are dysregulated in GBM samples as revealed by lipid profiling^11^, highlighting that targeting lipid metabolism might represent a promising approach in GBM therapy.

Lipoxygenases (LOXs) are a family of enzymes that produce oxylipin from polyunsaturated fatty acids^12,13^. Excessive oxylipin triggers ferroptosis, an iron- and lipotoxicity-dependent but caspase-independent form of regulated cell death distinct from apoptosis, necrosis, and autophagy^14^. The LOXs consist of six isoforms including ALOXE3, ALOX5, ALOX12, ALOX12B, ALOX15, and ALOX15B. Although the role of ALOXE3 in cancer development is yet to be defined, growing evidences revealed that the other LOXs members and their oxylipin products are key mediators for tumorigenesis in different types of cancers via diverse mechanisms. For instance, ALOX5 deficiency promotes lung cancer progression by suppressing T cell recruitment into the tumor microenvironment^15^. ALOX12B promotes cervical cancer development by inducing cancer cell proliferation^16^. Up-regulation of ALOX12-mediated 12-hydroxyeicosatetraenoic acids (12-HETE) triggers recurrence of non-alcoholic fatty liver disease associated hepatocellular carcinoma^17^. In addition, overexpressed ALOX12 in platelet induces angiogenesis and tumor growth in human prostate cancer^18^. On the contrary, ALOX12 has been shown to mediate p53-induced ferroptosis during lymphoma suppression^19^. Similarly, ALOX15 also plays dual roles in cancer development. On the one hand, ALOX15 promotes breast cancer progression by enhancing the invasion of tumor cells^20^. On the other hand, ALOX15 acts as a tumor suppressor in both colon cancer and prostate cancer^21,22^. Additionally, ALOX15B has been identified as a candidate diagnostic marker for non-small cell lung cancer^23^. Despite these promising findings, the understanding of LOXs and their oxylipin products in GBM development are still limited.

Considering these studies, the role of LOXs in GBM development remains elusive. In the present study, we aimed to determine the expression profiles and the regulatory roles of LOXs and their oxylipin metabolites in GBM development. We demonstrated that ALOXE3 expression is significantly decreased in human GBM. ALOXE3 deficiency triggers malignant GBM development through promoting cell survival and migration of GBM cells. On the one hand, ALOXE3 silencing renders resistance to p53-mediated ferroptosis of GBM cells. On the other hand, ALOXE3 knockdown leads to 12-HETE accumulation and release into the extracellular environment. Mechanistic studies showed that miR-18a directly targets ALOXE3 and suppresses its expression and activity in GBM cells. Furthermore, 12-HETE induces migration of GBM cells by activating PI3K-Akt pathway in an autocrine fashion. These findings highlighted an essential role of miR-18a/ALOXE3 axis in the regulation of GBM development, which may serve as a potential diagnostic and therapeutic target for GBM.

## Results

### Decreased ALOXE3 expression in human GBM

**Fig. 1:**
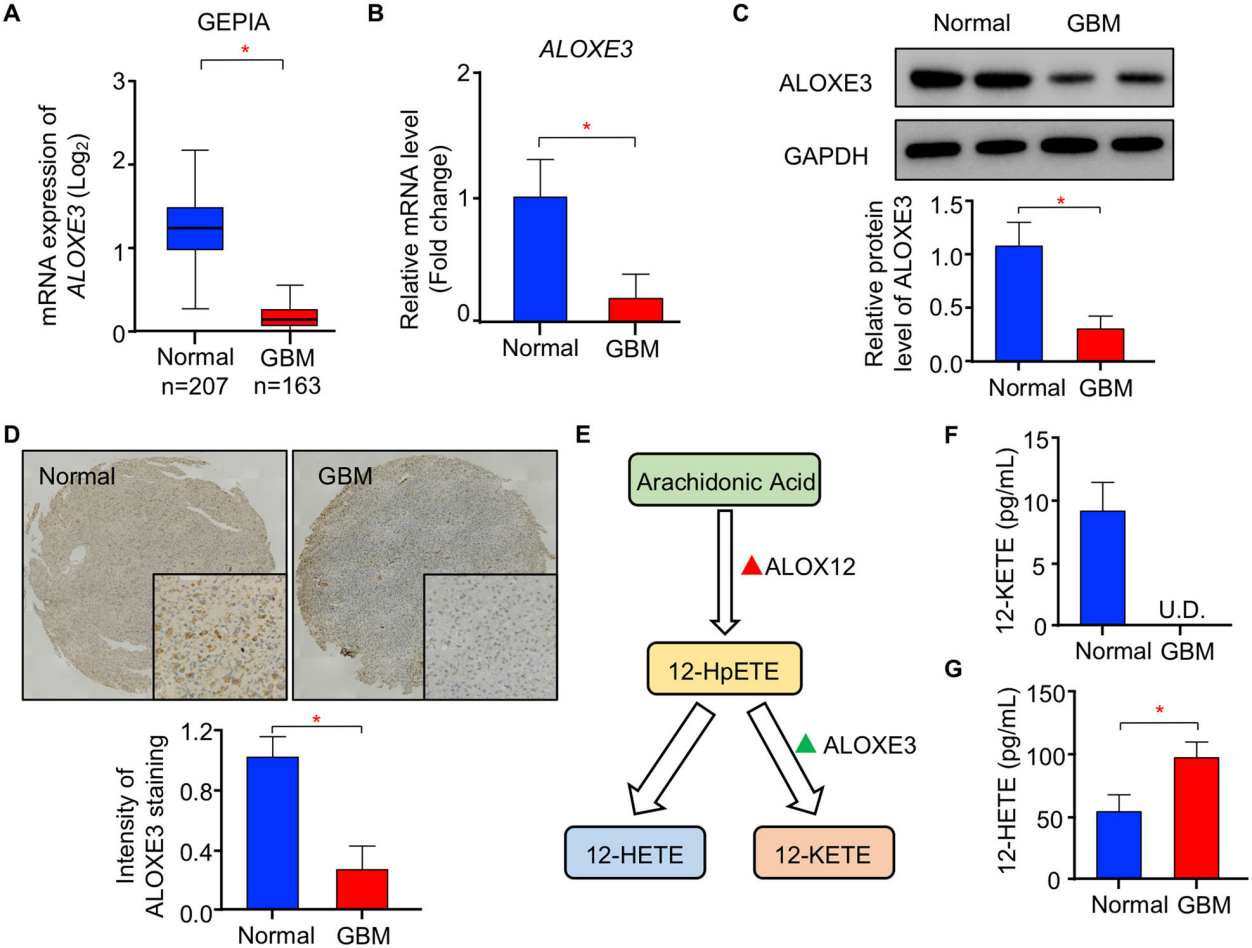
Expression of ALOXE3 is down-regulated in human GBM. **A** GEPIA analysis of ALOXE3 mRNA level in open access datasets (*n* = 207 for normal brain tissues and *n* = 163 for GBM). **B**, **C** Human GBM tissues and normal brain tissues were used (*n* = 6). **B** Relative mRNA level of *ALOXE3* normalized with *GAPDH* in human GBM tissues and normal brain tissues. The mRNA level is expressed as fold change over normal brain tissues. **C** Immunoblotting analysis of ALOXE3 and GAPDH in human GBM tissues and normal brain tissues. Representative immunoblot images are shown. The bar chart is the relative expression level of ALOXE3 normalized with GAPDH. **D** Immunohistochemistry staining of ALOXE3 in human GBM tissue microarray (*n* = 8 for normal brain tissue and *n* = 19 for GBM). **E** Diagram of arachidonic acid metabolism catalyzed by ALOXE3. **F**, **G** Circulating levels of (**F**) 12-KETE and (**G**) 12-HETE in the cerebrospinal fluid of GBM patients (*n* = 10). All data are represented as the mean ± s.e.m. *p < 0.05 (Student’s t-test).

To explore the pathophysiological relevance of LOXs in human GBM, we first investigated the expression of LOXs in open access databases via the Gene Expression Profile Interactive Analysis (GEPIA)^24^. Among the six members of LOXs family, only *ALOXE3* expression was significantly down-regulated in human GBM compared to normal brain tissues (Fig. 1A and Figure. S1A–E). To further validate the reduced expression of ALOXE3 in GBM, we analyzed the ALOXE3 levels in clinical human GBM lesions and normal brain tissues obtained from surgical trauma patients. Real-time quantitative PCR (QPCR) analysis showed that the mRNA abundance of *ALOXE3* was down-regulated in human GBM tissues when compared with normal brain tissues (Fig. 1B). Consistently, the protein level of ALOXE3 was decreased in GBM tissues as revealed by immunoblotting analysis in GBM samples and immunohistochemical (IHC) staining in tissue microarray (TMA; Fig. 1C–D). Since 12-hydroperoxyeicosatetraenoic acids (12-HpETE) metabolized from arachidonic acid, can be further catalyzed into 12-HETE or 12-ketoeicosatetraenoic acids (12-KETE) in an ALOXE3-dependent manner^25^ (Fig. 1E), we next explored the circulating levels of 12-HETE and 12-KETE in cerebrospinal fluid (CSF) of GBM patients and their controls. The 12-KETE was undetectable, while the 12-HETE level in CSF was elevated in GBM patients as a result of ALOXE3 down-regulation (Fig. 1F–G). Altogether, these findings suggested that ALOXE3 is significantly down-regulated in GBM and may play a role in GBM development.

### ALOXE3 deficiency promotes orthotopic GBM growth in immunodeficient nude mice

**Fig. 2:**
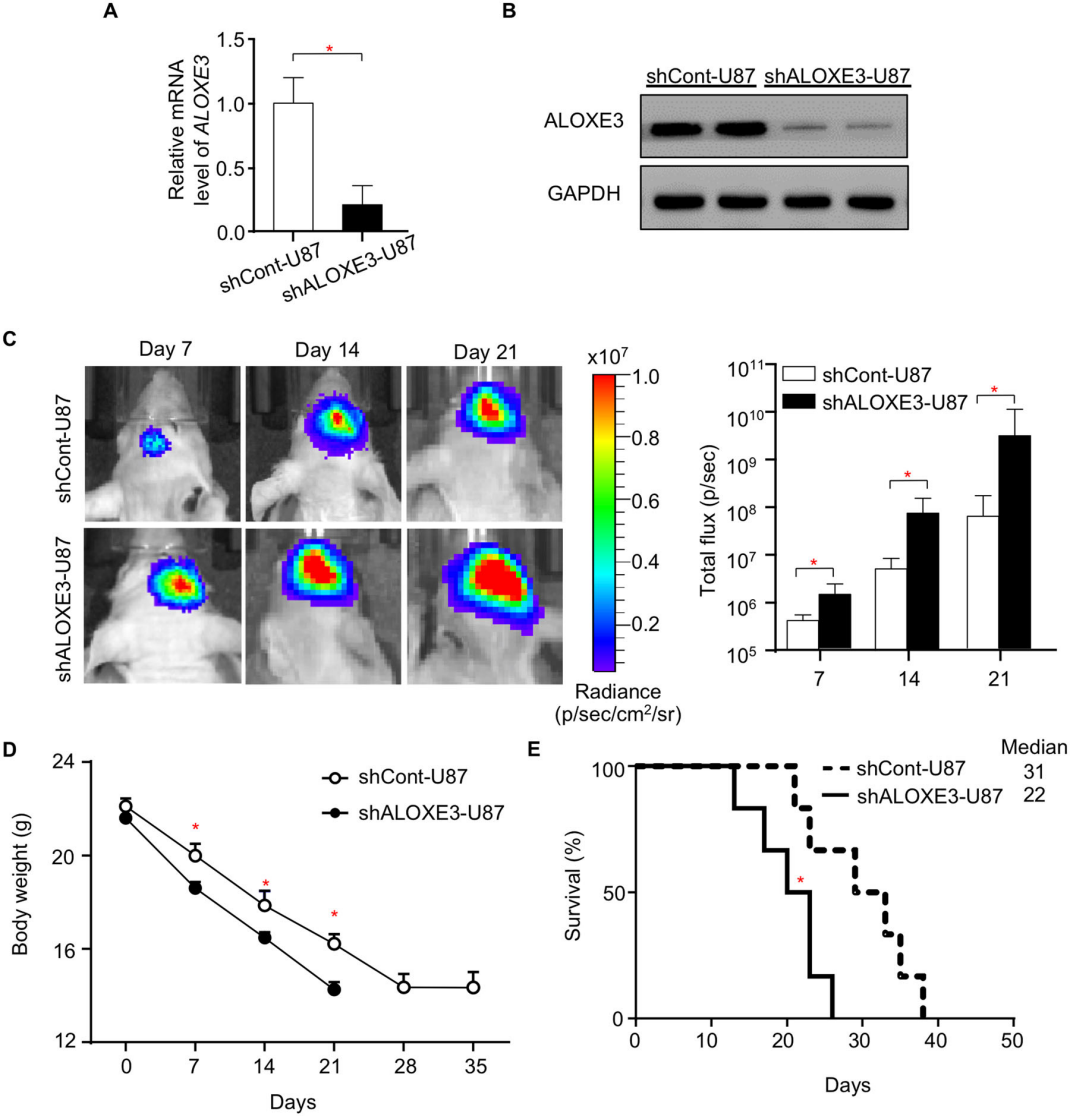
ALOXE3 deficiency promotes orthotopic GBM growth and shortens lifespan in mice. **A** Relative mRNA level of *ALOXE3* normalized with *GAPDH* in shALOXE3-U87 and shCont-U87 cells (*n* = 6). **B** Immunoblotting analysis of ALOXE3 and GAPDH in shALOXE3-U87 and shCont-U87 cells. Representative immunoblot images are shown (*n* = 6). **C**–**E** shALOXE3-U87 and shCont-U87 cells were implanted orthotopically into the hippocampus of immunodificient nude mice (*n* = 6). **C** In vivo bioluminescent imaging of nude mice at indicated time points. The right panel is the quantification of luminescence signal intensity. **D** Body weight of mice. All data are represented as the mean ± s.e.m. *p < 0.05 (Student’s t-test). **E** Survival curve of mice. Medians are shown. *p < 0.05 (Long-rank test).

To determine the role of ALOXE3 in the development of GBM, we employed a lentiviral mediated shRNA silencing approach to generate a U87 human GBM stable cell line with knockdown of ALOXE3. Three shRNAs were designed to target ALOXE3. Among these three shRNAs, shRNA2 exerted the greatest knockdown efficiency and was chosen for all subsequent experiments (shRNA2 was defined as shALOXE3; Figure. S2A–B). The ALOXE3 expression level was significantly down-regulated in U87 cells transduced with lentiviral shALOXE3 (so-called shALOXE3-U87 cells) when compared with its control cells (so-called shCont-U87 cells), as revealed by QPCR and Western blot analysis (Fig. 2A and B). These findings confirmed the successful knockdown of ALOXE3 in U87 GBM cells. Next, we generated orthotopic GBM-bearing mice by orthotopically implanting shALOXE3-U87 or shCont-U87 cells into the hippocampus of immunodeficient nude mice. We extracted the protein from the orthotopic tumor and confirmed the down-regulation of ALOXE3 (Figure. S3A–B). U87 cells transduced with lentiviral shALOXE3 promoted the tumor growth in nude mice as revealed by the in vivo bioluminescent imaging (Fig. 2C), suggesting a protective role of ALOXE3 in malignant progression of GBM. Consistently, shALOXE3-U87 cells accelerated body weight loss and shortened the lifespan of mice (Fig. 2D and E). Similarly, inhibitory effects of ALOXE3 on GBM were also observed in another well-established GBM cell line U251 (Figure. S4A–C). These results illuminated that ALOXE3 deficiency promotes the malignant progression of GBM in mice.

### ALOXE3 knockdown renders GBM cells resistant to p53-induced ferroptosis

**Fig. 3:**
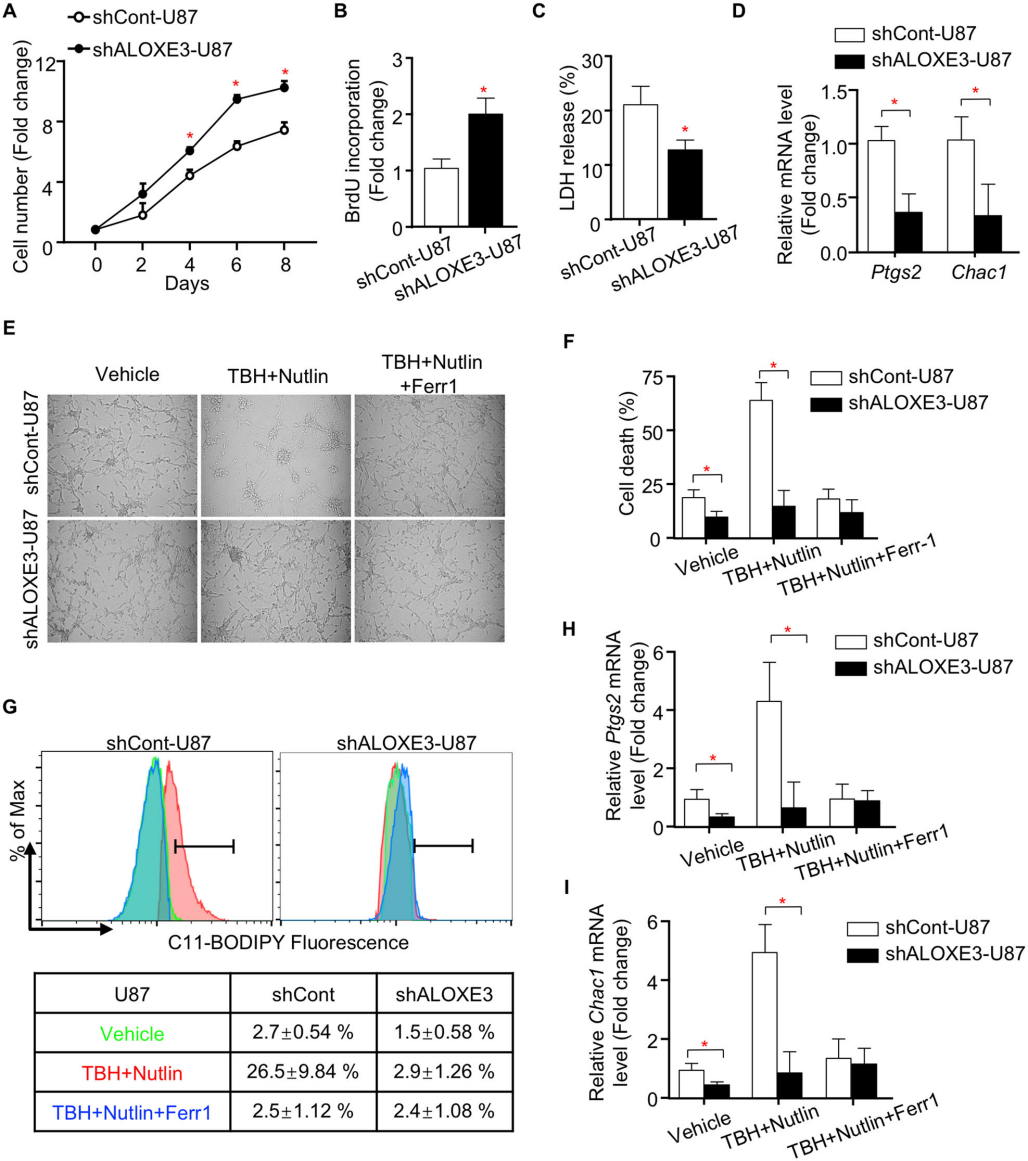
GBM cells with ALOXE3 silencing are resistant to p53-induced ferroptosis. **A**–**D** shALOXE3-U87 and shCont-U87 cells were used (*n* = 6). **A** Cell number was determined by trypan blue assay at indicated time points. **B** BrdU incorporation of cells. **C** LDH release of cells. **D** Relative mRNA levels of *Ptgs2* and *Chac1* normalized with *GAPDH* in cells. **E–I** shALOXE3-U87 and shCont-U87 cells were pre-treated with Nutlin-3a for 12 h and followed with TBH and Nutlin-3a ± Ferr1 treatment for additional 8 h (*n* = 6). **E** Representative images of cells. **F** Trypan blue staining for cell death. **G** C11-BODIPY staining for lipid peroxidation. **H**, **I** Relative mRNA levels of (**H**) *Ptgs2* and (**I**) *Chac1* normalized with *GAPDH* in cells. All data are represented as the mean ± s.e.m. *p < 0.05 (Student’s *t* test).

To further explore the underlying reason by which ALOXE3 silencing promotes GBM progression in mice, we investigated the cell proliferation and cell survival of shALOXE3-U87 cells. shALOXE3-U87 cells displayed higher proliferation rate while decreased cell death when compared with shCont-U87 cells (Fig. 3A–C). Apoptosis was not affected in GBM cells with ALOXE3 knockdown, as indicated by comparable activity and expression level of cleaved caspase 3 (Figure. S5A–D). Emerging evidences illustrated that LOXs catalyze H(p)ETEs formation from arachidonic acid, in turn contributing to the cellular accumulation of lipid hydroperoxides and initiating ferroptosis^26^. Thus, we next measured ferroptosis in shALOXE3-U87 cells and shCont-U87 cells. QPCR analysis of *Ptgs2* and *Chac1*, two well-known markers for ferroptosis, showed that the ferroptosis was significantly suppressed in shALOXE3-U87 cells (Fig. 3D). Consistent with our in vitro observation, *Ptgs2* and *Chac1* expression levels were also down-regulated in orthotopic tumors of mice implanted with shALOXE3-U87 cells (Figure. S3C). These findings indicated that ALOXE3 is involved in the regulation of ferroptosis.

Generally, ferroptosis can be generally classified into p53-mediated and acyl-CoA synthetase long-chain family member 4 (ACSL4)-dependent ferroptosis. We firstly ascertained whether p53-dependent ferroptosis was altered in U87 cells with ALOXE3 silencing. p53-mediated ferroptosis can be induced in shCont-U87 cells upon combined treatment with both Nutlin-3a (a molecule that activates p53 via blocking MDM2-p53 interaction) and Tert-Butyl hydroperoxide (TBH; Fig. 3E, F). However, p53-mediated ferroptosis was largely abrogated by ALOXE3 deficiency (Fig. 3E, F). Notably, shALOXE3-U87 cells showed a decreased accumulation of reactive oxidative species (ROS) upon combined treatment with both Nutlin-3a and TBH, as reflected by C11-BODIPY staining (Fig. 3G). Compared with shCont-U87 cells, increased expression of ferroptotic genes was not observed in shALOXE3-U87 cells upon Nutlin-3a and TBH treatment (Fig. 3H–I), indicating that GBM cells with ALOXE3 knockdown were resistant to p53-dependent ferroptosis. Furthermore, suppressed p53-mediated ferroptosis was also observed in U251 GBM cells (Figure. S6A–C). The mechanism of p53-dependent ferroptosis is distinct from ACSL4-dependent ferroptosis, which can be induced by Erastin or RSL3^19,27–30^. We observed that ferroptosis was induced by Erastin or RSL3 in both shALOXE3-U87 cells and shCont-U87 cells in a similar magnitude. The ferroptotic response was totally reversed by the ferroptosis inhibitor ferrostatin-1 (Ferr1; Figure. S7A–D). Collectively, these findings suggested that ALOXE3 is required for p53-mediated ferroptosis but is dispensable for ACSL4-dependent ferroptosis.

### p53 activates lipoxygenase activity of ALOXE3 through suppressing SLC7A11

**Fig. 4:**
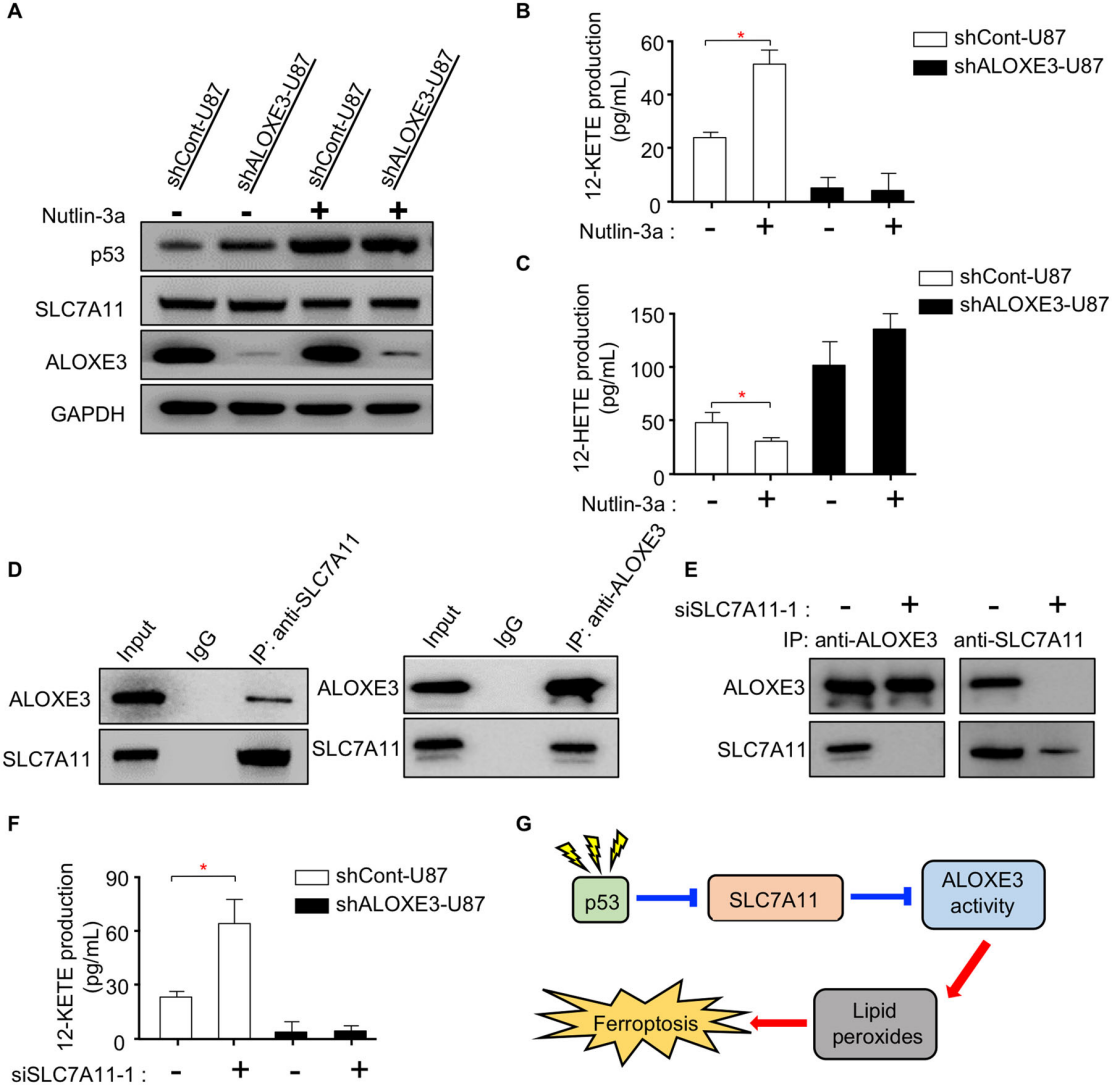
p53 activates lipoxygenase activity of ALOXE3 through suppressing SLC7A11. **A** Immunoblotting analysis of p53, SLC7A11, ALOXE3, and GAPDH in shALOXE3-U87 and shCont-U87 cells with or without Nutlin-3a treatment. Representative immunoblot images are shown (*n* = 6). **B**, **C** Levels of (**B**) 12-KETE and (**C**) 12-HETE in culture medium harvested from shALOXE3-U87 and shCont-U87 cells with or without Nutlin-3a treatment (*n* = 6). **D** Western blot analysis of the endogenous interaction between ALOXE3 and SLC7A11 in U87 cells. **E** Western blot analysis of the endogenous interaction between ALOXE3 and SLC7A11 in U87 cells treated with siSLC7A11-1 or siCont. **F** 12-KETE levels of culture medium harvested from shALOXE3-U87 and shCont-U87 cells transfected with siSLC7A11-1 or siCont (*n* = 6). **G** Mechanistic insight into p53-mediated activation of ALOXE3 activity and its associated ferroptosis. All data are represented as the mean ± s.e.m. *p < 0.05 (Student’s *t*-test).

Knowing that ALOXE3 is required for p53-mediated ferroptosis, we aimed to elucidate whether ALOXE3 is regulated by p53. Western blot analysis showed that Nutlin-3a dramatically up-regulated p53 level, whereas there was no obvious effect on ALOXE3 level upon p53 activation (Fig. 4A). Since the expression level of ALOXE3 was not affected in response to p53 activation, we next examined whether ALOXE3 activity is modulated by p53. The lipoxygenase activity of ALOXE3 was determined by measuring the 12-KETE and 12-HETE production of U87 GBM cells. Interestingly, the ALOXE3 activity was elevated in shCont-U87 cells upon p53 activation, as revealed by up-regulated 12-KETE but down-regulated 12-HETE production (Fig. 4B–C). These findings indicated that p53 modulates ALOXE3 lipoxygenase activity. Thus, we explored whether p53 modulates ALOXE3 activity through SLC7A11, a well-known p53 down-stream target involved in ferroptosis^19^. SLC7A11 was negatively regulated by p53 in U87 GBM cells, as indicated by western blot (Fig. 4A). Co-immunoprecipitation analysis revealed that there was an interaction between SLC7A11 and ALOXE3 (Fig. 4D). To further elucidate the regulatory role of SLC7A11 on ALOXE3 activity, we employed siRNA to silence SLC7A11 in U87 GBM cells. Three distinct siRNAs against SLC7A11 (siSLC7A11) were evaluated. siSLC7A11-1 achieved the highest knockdown efficiency and was selected for subsequent experiments (Figure. S8A-B). Of note, the addition of siSLC7A11-1 disrupted the ALOXE3-SLC7A11 complex (Fig. 4E). Furthermore, siSLC7A11-1-mediated silencing of SLC7A11 effectively promoted ALOXE3 activity in U87 GBM cells (Fig. 4F and Figure. S8C), illustrating that SLC7A11 is a negative regulator of ALOXE3 activity. Together, these observations suggested that p53 can indirectly promote ALOXE3 activity by negatively regulating SLC7A11 (Fig. 4G).

### miR-18a directly targets ALOXE3 and suppresses its expression in GBM cells

**Fig. 5:**
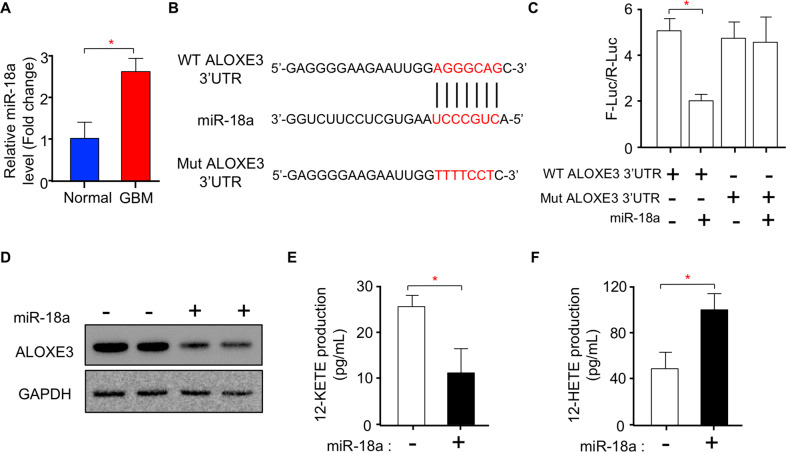
miR-18a directly targets ALOXE3 and suppresses its expression in GBM cells. **A** Relative miR-18a level normalized with U6 in human GBM tissues and normal brain tissues (*n* = 6). **B** Target sequence of miR-18a in the wildtype (WT) ALOXE3 3’UTR and sequence of mutated (Mut) ALOXE3 3′UTR. **C** Measurement of firefly-luciferase activity normalized with the renilla-luciferase activity in U87 cells (*n* = 6). **D** Immunoblotting analysis of ALOXE3 and GAPDH in U87 cells with miR-18a mimic or mimic-negative control treatment. Representative immunoblot images are shown (*n* = 6). **E-F** Levels of (**E**) 12-KETE and (**F**) 12-HETE in culture medium harvested from U87 cells with miR-18a or mimic-negative control treatment (*n* = 6). All data are represented as the mean ± s.e.m. *p < 0.05 (Student’s t-test).

miRNAs have been reported to regulate tumor-associated proteins in multiple cancers^31,32^. Thus, we next ascertained whether ALOXE3 down-regulation in GBM is mediated by miRNAs. Consistent with previous findings^33,34^, the level of miR-18a was significantly up-regulated in human GBM tissues (Fig. 5A). Using in silico analysis, we identified a potential miR-18a targeting sequence in the 3′UTR of ALOXE3 (Fig. 5B). To verify whether miR-18a regulates ALOXE3 expression by binding to its 3′UTR, we mutated 3′UTR of ALOXE3 and performed a firefly/renilla dual-luciferase reporter assay (Fig. 5B–C). Results from the luciferase reporter assay showed that miR-18a decreased the luciferase activity in U87 cells transfected with wildtype (WT) ALOXE3 3′UTR, but not in cells transfected with mutated (Mut) ALOXE3 3′UTR (Fig. 5C). Upon miR-18a treatment, the protein level of ALOXE3 was dramatically down-regulated in U87 cells coupled with decreased 12-KETE and increased 12-HETE production (Fig. 5D–F). Taken together, these findings demonstrated that miR-18a directly targets ALOXE3 and suppresses its expression and activity in GBM cells.

### ALOXE3 silencing promotes the migration of GBM cells via 12-HETE in an autocrine manner

**Fig. 6:**
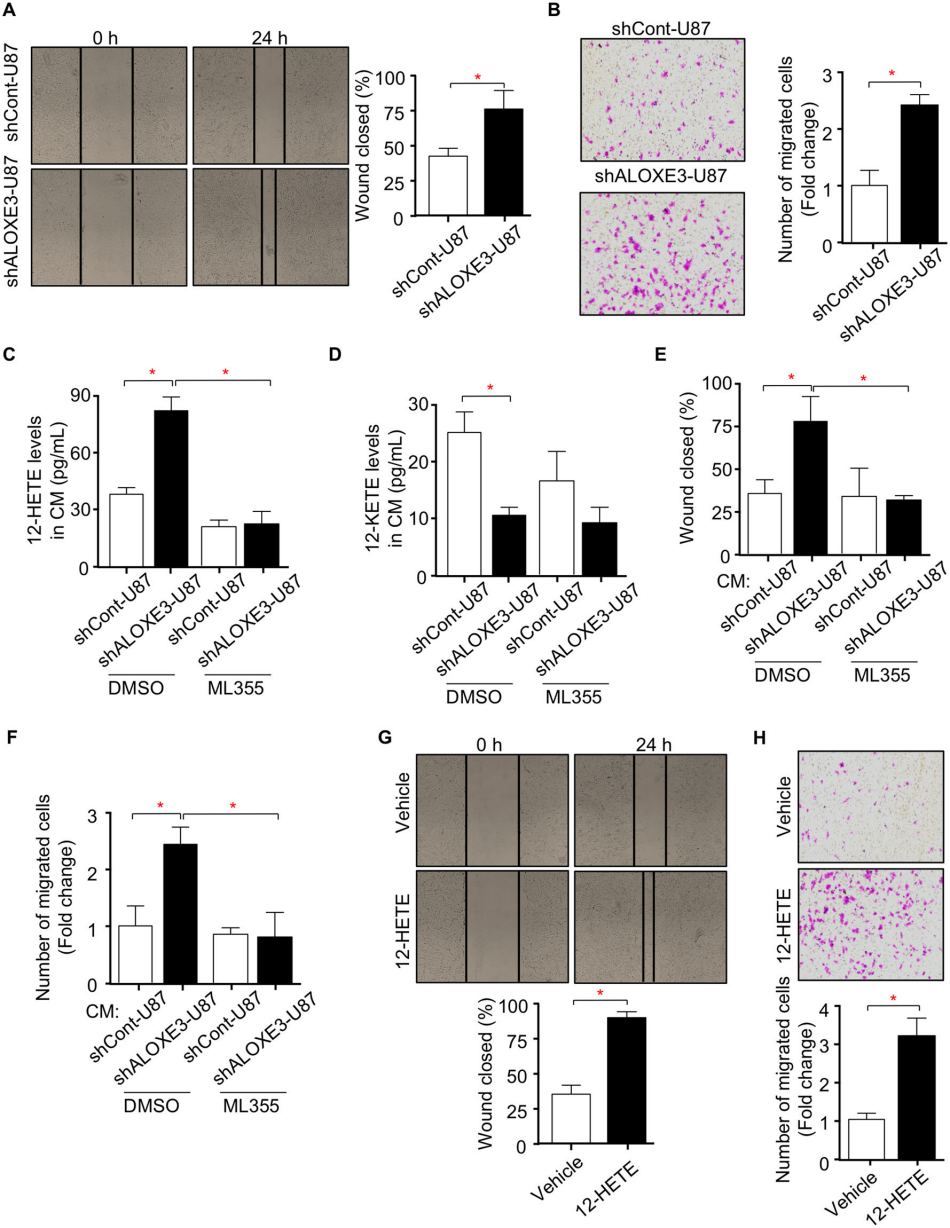
Knockdown of ALOXE3 promotes migration of GBM cells via 12-HETE in an autocrine manner. **A** Wound healing assay of shALOXE3-U87 and shCont-U87 cells was determined at 0 and 24 h after the wound was created. The right panel is the percentage of the wound closed at 24 h (*n* = 6). **B** Transwell assay of shALOXE3-U87 and shCont-U87 cells. The right panel is the quantification of the number of migrated cells (*n* = 6). **C**, **D** Levels of (**C**) 12-HETE and (**D**) 12-KETE in conditional medium (CM) harvested from shALOXE3-U87 and shCont-U87 cells treated with ML355 or DMSO as control (*n* = 6). **E**, **F** U87 cells were incubated with CM harvested from shALOXE3-U87 and shCont-U87 cells treated with ML355 or DMSO as vehicle control, and followed with (**E**) wound healing assay and (**F**) transwell assay (*n* = 6). **G**, **H** U87 cells were treated with 12-HETE or Vehicle control, and followed with (**G**) wound healing assay and (**H**) transwell assay (*n* = 6). All data are represented as the mean ± s.e.m. *p < 0.05 (Student’s t-test).

Increased migration capacity of GBM cells is also pivotal for GBM development, we next examined whether knockdown of ALOXE3 in GBM cells has any effect on migration. Wound healing and transwell assays illustrated that U87 and U251 GBM cells with ALOXE3 knockdown displayed increased migration capacity when compared to control cells (Fig. 6A, B and Figure. S9A-B), indicating that ALOXE3 down-regulation promotes migration of GBM cells. Since 12-HETE can modulate cell metabolism and functions in an autocrine or paracrine manner^17,25,35,36^, we hypothesized that ALOXE3 knockdown in GBM cells promoted secretion of 12-HETE, which in turn enhanced migration of GBM cells in an autocrine manner. To investigate this assumption, we collected conditional medium (CM) from the U87 GBM cells with ALOXE3 silencing, and measured the 12-HETE levels. As a result of ALOXE3 knockdown, the extracellular 12-HETE level was significantly elevated in CM of U87 GBM cells transduced with lentiviral shALOXE3 or transfected with siALOXE3 (Fig. 6C and Figure. S10A-D). U87 GBM cells were incubated with CM harvested from shALOXE3-U87 cells or shCont-U87 cells, and followed with wound healing and transwell assays. Cell migration capacity of U87 GBM cells was stimulated by the CM obtained from shALOXE3-U87 cells when compared with CM from shCont-U87 cells (Fig. 6E, F). We next explored whether blocking 12-HETE production by ALOX12 inhibitor ML355, can inhibit such effect. Results showed that ML355 effectively down-regulated the 12-HETE secretion but with no obvious effect on 12-KETE secretion of shALOXE3-U87 and shCont-U87 cells (Fig. 6C–D). ML355 completely reversed the migration-promoting effect of CM derived from shALOXE3-U87 cells (Fig. 6E, F).

To further elucidate whether 12-HETE exerts migration-promoting effect, GBM cells were treated with 12-HETE and subjected to wound healing and transwell assays. 12-HETE significantly induced the migration capacity of GBM cells (Fig. 6G, H), suggesting that 12-HETE mediates the migration-promoting effect of shALOXE3-U87 cells. Matrix metalloproteins (MMPs) and Ezrin-Radixin-Moesin (ERM) are two families contributing to cell migration during tumor malignant development^5,37,38^. We found out that MMPs (MMP2, MMP7, MMP9, and MMP13) but not ERM family were up-regulated in U87 GBM cells with 12-HETE stimulation (Fig. S11A). Consistent with our in vitro observation, ML355 effectively down-regulated the 12-HETE level while had no obvious effect on 12-KETE level in the tumor of mice orthotopically implanted with shALOXE3-U87 cells (Fig. S11B–C). Consequently, ML355 largely rescued the migration-promoting effects of shALOXE3-U87 cells in mice, as indicated by the decreased mRNA abundance of *MMPs* (Fig. S11D–G). Although ferroptosis is associated with the accumulation of oxylipin, the product of LOXs catalysis, we found out that 12-HETE is dispensable for p53-induced ferroptosis in U87 GBM cells (Fig. S12A–D). These data indicated that GBM cells with ALOXE3 deficiency promotes migration of GBM cells via 12-HETE in an autocrine manner.

### 12-HETE induces migration of GBM cells via activating GsPCR-PI3K-Akt pathway

Recent studies have shown that 12-HETE promoted MMPs expression by activating PI3K-Akt pathway^17,36^. Therefore, we next investigated if PI3K-Akt pathway was activated in U87 GBM cells upon 12-HETE treatment. Results showed that 12-HETE promoted expression and phosphorylation of PI3K, and subsequently stimulated the Akt phosphorylation at Thr308 in U87 GBM cells (Fig. S13A), illustrating that 12-HETE significantly activated PI3K-Akt pathway. 12-HETE has been reported to exert its effects through stimulation of G-protein-coupled receptors (GPCRs)^17,36^. GPCRs consists of 3 different subtypes, including G_q_/11PCR, G_i_PCR and G_s_PCR^35^. To identify which subtype of GPCRs mediates the effect of 12-HETE on promoting migration, U87 GBM cells were pre-treated with inhibitors of 3 different subtypes of GPCRs and followed by wound healing assay. Pretreatment with the G_q_/11PCR or G_i_PCR inhibitors, YM254890 and pertussis toxin, did not influence the 12-HETE-mediated migration-promoting effect (Fig. S13B–C). However, G_s_PCR inhibitor melittin completely blunted the 12-HETE-mediated PI3K-Akt pathway activation and migration-promoting effect on GBM cells (Fig. S13D–G). Taken together, these results showed that 12-HETE induces migration through activating G_s_PCR-PI3K-Akt pathway in an autocrine manner.

## Discussion

Emerging evidences revealed that LOXs mediate the development of cancer via multiple pathways. However, the expression and functional roles of LOXs in GBM is still unclear. Our study demonstrates that ALOXE3 is dramatically down-regulated in GBM. Knockdown of ALOXE3 in GBM cells promotes orthotopic tumor growth in mice. The tumor-promoting role of ALOXE3 deficiency is owing to its resistance to p53-dependent ferroptosis and potentiating effect on migration capacity of GBM cells. Mechanistically, miR-18a directly targeted ALOXE3 and suppressed its expression and functions in GBM cells. In addition, ALOXE3 deficiency is shown to promote migration of GBM cells via 12-HETE in an autocrine manner. This is the first study uncovering that the miR-18a/ALOXE3 axis plays an important role in regulating the cell survival and migration capacity of GBM.

LOXs have been illustrated to play crucial roles in ferroptosis, a recently characterized cell death that is triggered by the accumulation of LOXs catalyzed products: oxylipin^26^. Ferroptosis is primarily regulated by GPX4 in an ACSL4-dependent manner. Several studies illuminated that LOXs-mediated generation of oxylipin is required for the ferroptosis induced by Erastin and RSL3^39,40^. Pharmacological inhibition or siRNA-mediated knockdown of ALOX5, ALOX15, or ALOX15B effectively prevented Erastin- and RSL3-induced ferroptotic cell death^39,40^. Role of ALOXE3 or ALOX12 in ferroptosis induced by Erastin and RSL3 are still unclear. Recently, Gu et al. identified a p53-mediated but not ACSL4-independent ferroptosis. ALOX12 was shown to be a key regulator for p53-mediated ferroptosis during lymphoma suppression^19^. However, our study finds out that the expression level of ALOXE3 but not ALOX12 is altered in GBM. In vitro study demonstrates that ALOXE3 is essential for p53-dependent ferroptosis but not ferroptosis induced by Erastin and RSL3 in GBM. In addition, we illuminate that p53 promotes ALOXE3 activity indirectly by transcriptionally inhibiting SLC7A11, uncovering the importance of ALOXE3 in p53-mediated tumor suppression via ferroptosis pathway.

MMPs and ERM are key regulators contributing to cell migration during tumor malignant development^5,37,38^. 12-HETE, an ALOX12 catalyzed oxylipin metabolite, has been shown to induce the expression of MMPs by activating GPCR-PI3K-Akt pathway^17,36^. Although the main oxylipin products of ALOXE3 is 12-KETE, our study shows that ALOXE3 silencing leads to 12-HpETE accumulation, in turn promoting 12-HETE production and secretion. In vitro, up-regulated MMPs are observed in GBM cells supplemented with 12-HETE. Mechanistically, 12-HETE promotes MMPs expression by activating G_s_PCR-PI3K-Akt pathway in an autocrine manner. We believe that the ALOXE3 inhibits GBM progression via two distinct pathways. On the one hand, ALOXE3 promotes cell death of GBM cells via the activation of ferroptosis. On the other hand, ALOXE3 decreases 12-HETE level by catalyzing 12-HpETE into 12-KETE, ameliorating the migration promoting effect of 12-HETE. It is also worthy to mention that the ALOXE3 related oxylipin 12-HETE has no effect on the ferroptosis of GBM cells.

GEPIA using open-access databases reveals that the ALOXE3 expression is down-regulated, whereas the ALOX5 is dramatically up-regulated in human GBM when compared with normal brain tissues. In this study, we focused on the regulatory role of ALOXE3 in GBM development mainly due to the role of ALOXE3 in cancer development is yet to be defined. Indeed, our team has already initiated a study to determine the role of ALOX5 in GBM development. In contrast to ALOXE3, ALOX5 exerts no effect on ferroptosis in GBM cells (Data not shown).

To rule out the possibility of off-target effects of shALOXE3, we employed two sets of siRNAs targeting ALOXE3 (siALOXE3) to inspect their effects on GBM cell function (Figure. S10A–B). Consistently, 12-HETE and 12-KETE secreted from U87 GBM cells transfected with siALOXE3 were up- and down-regulated, respectively (Figure. S10C-D). In addition, U87 GBM cells transfected with siALOXE3 were resistant to p53-mediated ferroptosis and enhanced migration in a 12-HETE dependent manner (Figure. S14A–E). Therefore, the GBM-promoting effects of shALOXE3-U87 cells are not mediated by off-target effects.

In summary, our study is the first to reveal that ALOXE3 is markedly decreased in GBM. ALOXE3 down-regulation promotes GBM development by inhibiting ferroptosis and enhancing migration of GBM cells. ALOXE3 is directly targeted and suppressed by miR-18a in GBM. Therefore, our findings uncover miR-18a/ALOXE3 axis as a potential therapeutic target for the treatment of GBM.

## Materials and methods

### Animal studies

Experiments were conducted on male NOD/SCID nude mice, which were obtained from Gempharmatech Company. All mice had free access to sterilized water and standard chow and were housed in a room with 23 °C temperature and 12 h light/dark cycle control. The 8-week-old male nude mice were randomly divided into two groups (6 mice per group). The investigators were not blinded to the experimental groups. Orthotopic implantation of GBM cells into the hippocampus of nude mice was performed as we previously described (*n* = 6)^5^. Mice were intraperitoneally injected with luciferin (150 mg/kg; catalog #P1043; Promega) and subjected to IVIS Spectrum in vivo imaging system (PerkinElmer) to determine the intracranial tumor size. All animal protocols were approved by the Animal Experimentation Ethics Committee, Southern Medical University.

### Real-Time QPCR

Total RNA was extracted from frozen tissues and cells using TRIzol (catalog #15596018; Thermo Fisher Scientific). For mRNA expression analysis, reverse transcription was performed with the PrimeScript RT reagent kit (catalog #RR037A; Takara). For miRNA expression analysis, reverse transcription was performed with the Taqman MicroRNA RT kit (catalog #4366596; Thermo Fisher Scientific). QPCR was done with Real-time PCR System (Applied Biosystems 7500) using SYBR Green (catalog #AQ131-02; Trans) and the specific primers (Table S1 and Table S2).

### Immunoblotting

Tissues or cell lines were prepared with a RIPA lysis buffer (catalog #9803; Thermo Fisher Scientific) containing protease inhibitor cocktail (catalog #DI101-01; Trans), and were homogenized by ultrasound sonication. Proteins were separated by SDS-PAGE electrophoresis and were transferred onto polyvinylidene difluoride membranes. Membranes were incubated with primary antibodies, followed by incubation with horseradish peroxidase (HRP)-conjugated secondary antibodies. The protein bands were visualized by an imaging system (Bio-Rad ChemiDoc^TM^ Imaging System) and quantified using ImageJ software. Antibodies for immunoblotting are shown in Table S3. Full images of immunoblotting are shown in Fig. S15.

### TMA construction and IHC staining

TMA containing human GBM lesions (*n* = 19) and normal brain tissue (*n* = 8) was generated by Servicebio company. All clinical human samples were collected from 2017 to 2019 in Yijishan Hospital and Shenzhen Hospital of Southern Medical University approved by their Human Ethics Committees. Samples from patients who received preoperative radiation or chemotherapy were excluded. Informed consent was obtained from all subjects. The tumor grading was defined by the Department of Pathology in Yijishan Hospital and Shenzhen Hospital of Southern Medical University. IHC staining was performed as we previously described^5^. Anti-ALOXE3 antibody (1:100; catalog #NBPI-32533; Novus) was used. The staining results were assessed by two independent investigators blinded to patients’ information.

### Measurement of 12-HETE and 12-KETE levels

Lumbar puncture was performed in GBM patients and their controls to collect the CSF. The levels of 12-HETE and 12-KETE in human CSF and CM of shALOXE3-U87 and shCont-U87 cells were determined by using UHPLC-QQQ-MS (Agilent 1290-6460). 12-HETE and 12-KETE were extracted with 200 μL mixture of methanol/water 1:1 (v/v). 12-HETE-d8 (catalog #334570; Cayman) was added as an internal standard.

### Generation of stable cell lines with ALOXE3 silencing

ALOXE3 shRNA lentiviral expression vector and its control vector were generated by Gene Chem using GV248 vector. Sequences for shRNAs are shown in Table S4. Lentiviral package was performed as we previously described^5^. At 16 h after transfection, the medium was replaced with a fresh complete medium. Lentivirus-containing supernatant was harvested at 2 days and 3 days after transfection. U87 and U251 cells were used for lentiviral transduction. All cell lines were mycoplasma-free and have been authenticated using short tandem repeat profiling. shALOXE3-U87/U251 cells were generated by incubating U87/U251 cells with shALOXE3 lentiviral supernatant in the presence of HitransG P (catalog #REVG005; Gene Chem) for 2 days, and followed by puromycin (catalog #A1113803; Thermo Fisher Scientific) selection for another 12 days.

### siRNA and miRNA transfection

U87 cells were seeded in 12-well plates and transfected with siRNAs or miRNAs for 48 h using Opi-MEM and lipofectamine RNAiMAX (catalog #13778150; Thermo Fisher Scientific). The siRNA against SLC7A11, its control siRNA, miR-18a mimic, and mimic-negative control were synthesized and purified by Sango Biotech. Sequences are shown in Table S5.

### Cell culture

To block the 12-HETE production, cells were treated with ML355 (10 μM; catalog #S6557; Selleckchem) for 24 h prior to CM collection. For 12-HETE treatment, cells were treated with 12-HETE (0.5 μM; catalog #S34550; Cayman) for 24 h. For GPCRs inhibition, cells were incubated with YM254890 (10 μM; catalog #29735; Cayman), Pert. Toxin (0.1 μM; catalog #P7208; Sigma) or Melittin (5 μM; catalog #B6628; APExBIO) for 12 h. For p53-dependent ferroptosis, U87 cells were pre-treated with Nutlin-3a (10 μM; catalog #A3671; APExBIO) for 12 h and followed with TBH (300 μM; catalog #B802372; Macklin) and Nutlin-3a (10 μM) ± Ferr1 (2 μM; catalog #A4371; APExBIO) treatment for additional 8 h. For ferroptosis induced by Erastin or RSL3, U87 cells were treated with Erastin (10 μM; catalog #B1524; APExBIO) or RSL3 (1 μM; catalog #B6095; APExBIO) ± Ferr1 (2 μM) treatment for 12 h.

### Measurement of caspase 3 activity and lipid peroxidation

2 × 10^5^ cells/well were seeded in 6-well-plate. The next day, activity of caspase 3 was determined by fluorometric assay kit according to the manufacturer’s protocol (*n* = 6). For lipid peroxidation staining (*n* = 6), ferroptosis was induced as indicated. After induction of ferroptosis, culture medium was removed, and the cells were harvested by trypsinization. Cells were resuspended in 500 μL PBS containing BODIPY C11 (2 μM; catalog #D3861; ThermoFisher). The stained cells were subjected to flow cytometry analysis using Sony SA3800 analyzer. The experiment was repeated three times.

### Co-immunoprecipitation

Co-immunoprecipitation was performed using Pierce^TM^ Classic Magnetic Co-IP kit (catalog #88804; Thermo Fisher). In brief, U87 cells were lysed by adding ice-cold IP lysis/wash buffer. To prepare immune complex, 500 μg total cell lysate was incubated with SLC7A11, ALXOE3 antibody, or rabbit IgG at 4 °C overnight. The immune complex was incubated with pre-washed pierce protein A/G magnetic beads at room temperature for an hour, followed by low-pH elution and immunoblotting analysis of ALOXE3 and SLC7A11.

### Luciferase reporter assay

The luciferase reporter constructs were generated by inserting WT ALOXE3 3′UTR or Mut ALOXE3 3′UTR into pGL3 luciferase vector. U87 cells were transfected with indicated luciferase vectors and miRNAs using lipofectamine 3000 reagent (catalog #L3000015; Thermo Fisher Scientific). 48 h after transfection, firefly/renilla dual luciferase activity was determined by luciferase reporter assay kit (catalog #FR201; Trans) as we previously described (*n* = 6)^5^.

### Wound healing assay

Cells were seeded in 12-well plate (1 × 10^5^ cells/well) containing DMEM with 10% FBS and 1% penicillin-streptomycin. The wound was created in the center of each well using a P20 pipette tips when the cell monolayer was formed. Images of cells were recorded at 0 h and 24 h post scratching. Wound closed at 24 h was calculated relative to 0 h, which was defined as 100% (*n* = 6). The experiment was repeated three times.

### Transwell migration assay

Transwell migration of cells was analyzed using 24-well transwell inserts (catalog #CLS3464; Corning) with 8.0 μm polyethylene terephthalate membrane. In brief, cells were seeded on the top chambers of transwell inserts with FBS-free DMEM. Lower chambers containing DMEM with 10% FBS and 1% penicillin-streptomycin allowed the cells to migrate through polyethylene terephthalate membrane. After 24 h, migrated cells on lower chambers were fixed with 100% methanol for 15 min, stained with 1% crystal violet (catalog #C6158; Sigma) in 20% methanol for 15 min at room temperature and followed by microscopy analysis (*n* = 6).

### Statistics

Sample size was determined based on previous publication and the variability observed in preliminary experiments. All data are shown as mean ± standard error of the mean (SEM). All statistical analysis was performed using SPSS or GraphPad Prism 8.0. Levene test was performed to assess the equality of variance. Unpaired Student *t-*test was done to compare two groups. One-way ANOVA with Bonferroni correction for multiple comparisons was used to compare more than two groups. The statistical significance for survival curve was calculated using long-rank test. Significance was set at *p* < 0.05 in all statistical comparisons.

## Supplementary information

Supplementary Figures and Tables

STR for U87 cells

STR for U251 cells
