# Supplementary material for: miR-18a promotes glioblastoma development by down-regulating ALOXE3-mediated ferroptotic and anti-migration activities
Source: Oncogenesis. 2021 Feb 12;10(2):15. doi: 10.1038/s41389-021-00304-3 (PMC7881152; doi:10.1038/s41389-021-00304-3)
Supplement: Supplementary file 1 — Supplementary Figures and Tables [file 41389_2021_304_MOESM1_ESM.pdf]

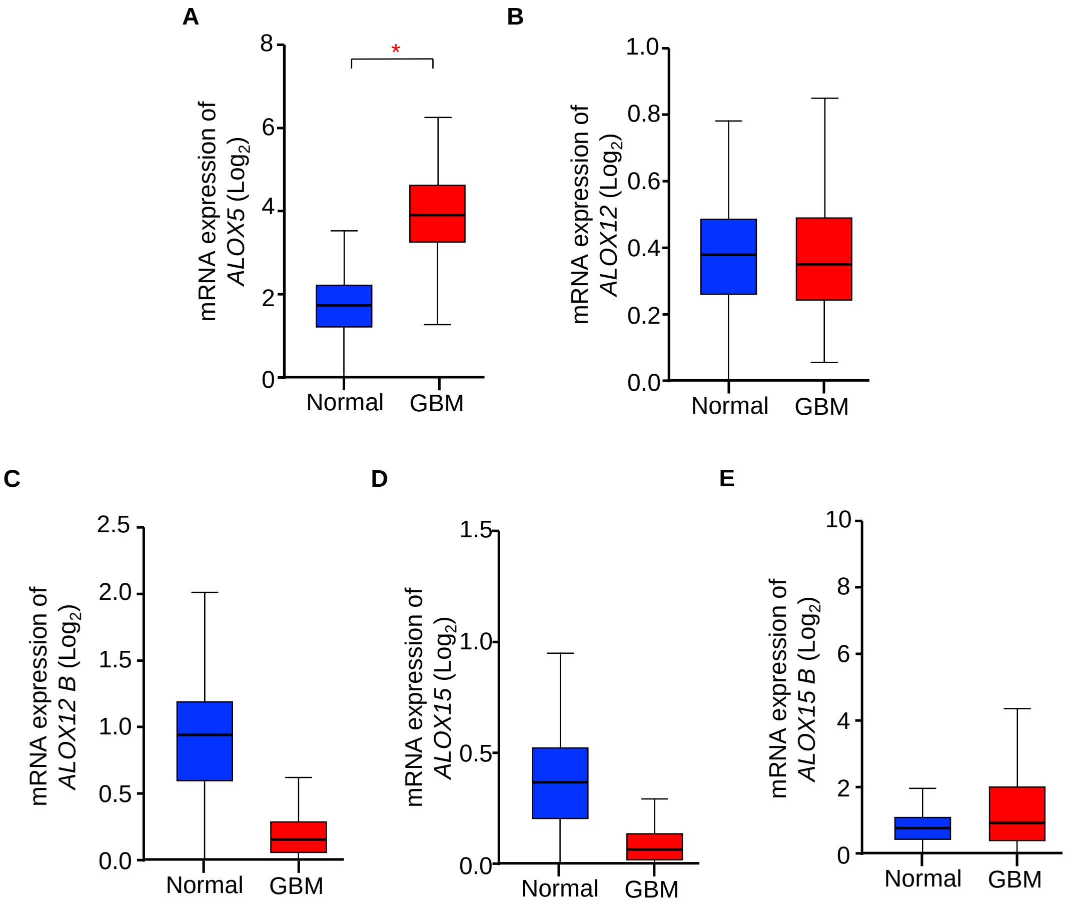

Figure S1. Expression of LOXs in human GBM.
Gene expression profile interactive analysis of relative mRNA levels of (A) *ALOX5*, (B) *ALOX12*, (C) *ALOX12B*, (D) *ALOX15* and (E) *ALOX15B* in human normal brain tissues and GBM (n=207 for normal brain tissue and n=163 for GBM). *p<0.05, (Student's t test).

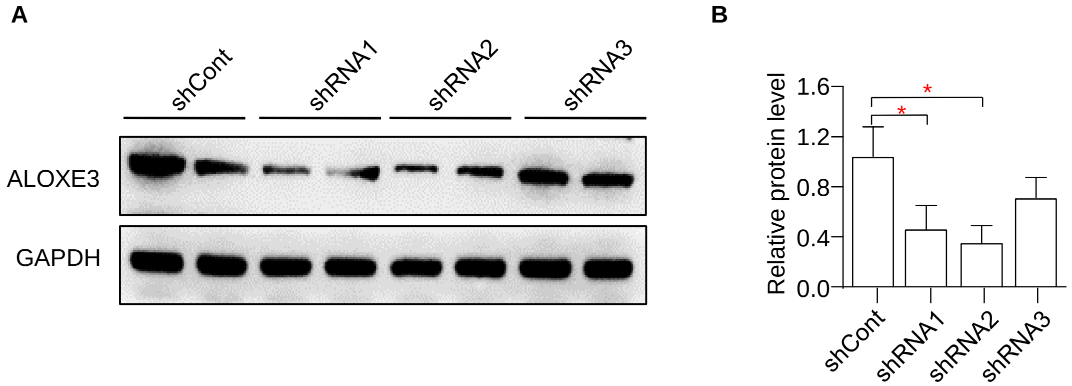

Figure S2. Knockdown of ALOXE3 by shRNAs in U87 GBM cells.

(A) Immunoblotting analysis of ALOXE3 and GAPDH in U87 cells expressing shRNAs targeting human ALOXE3. Representative images are shown. (B) The bar chart is relative expression level of ALOXE3 normalized with GAPDH (n=6). All data are represented as the mean±s.e.m. *p<0.05 (Student's t test).

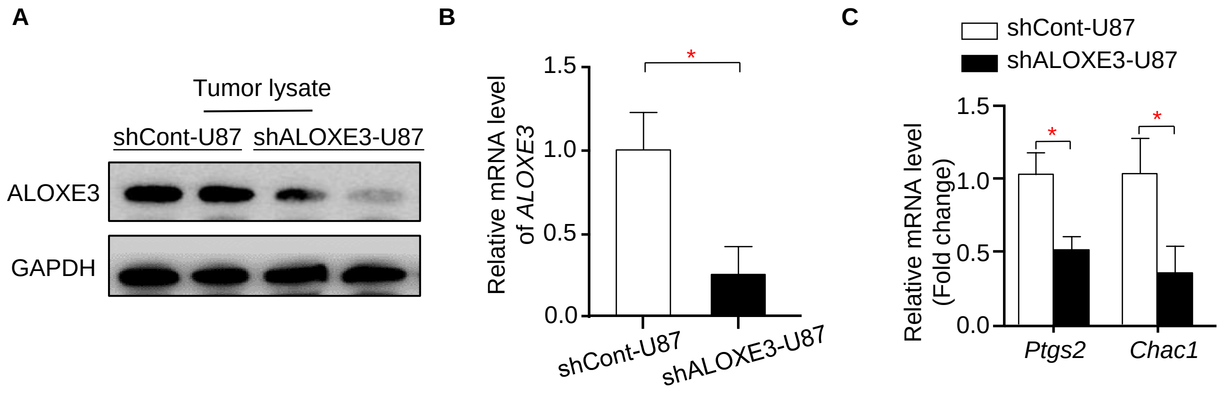

Figure S3. ALOXE3 and ferroptotic genes are significantly decreased in tumors harvested from mice orthotopically implanted with shALOXE3-U87 cells.

Orthotopic tumors isolated from hippocampus of immunodeficient mice were used (n=6). (A) Immunoblotting analysis of ALOXE3 and GAPDH in orthotopic tumors. Representative immunoblot images are shown. (B) Relative mRNA level of *ALOXE3* normalized with *GAPDH* in orthotopic tumors. (C) Relative mRNA levels of *Ptgs2* and *Chac1* normalized with *GAPDH* in orthotopic tumors. All data are represented as the mean ± s.e.m. *$p<0.05$ (Student's t test).

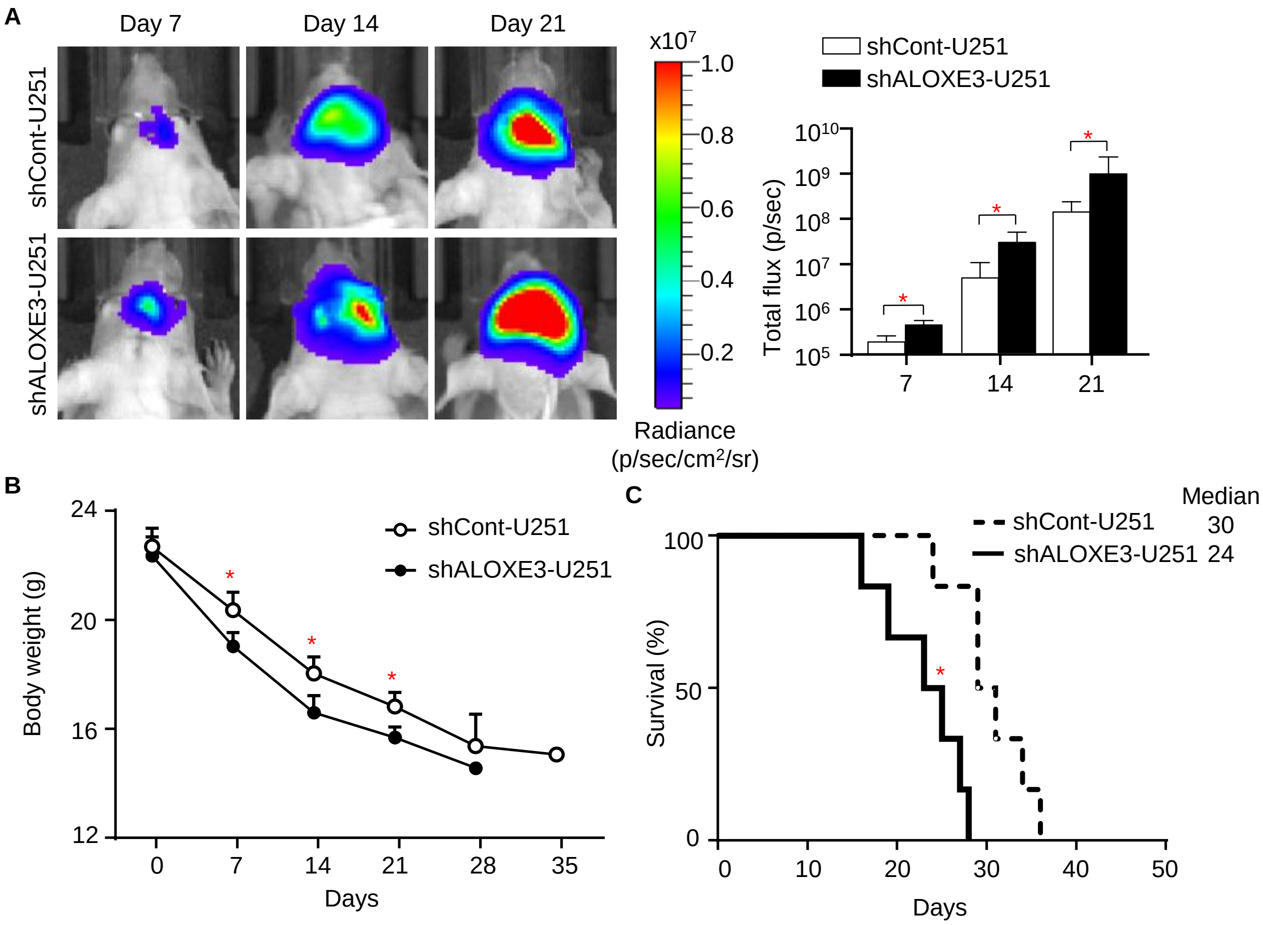

Figure S4. Inhibitory effects of ALOXE3 on GBM were observed in U251 GBM cells. shALOXE3-U251 and shCont-U251 cells were implanted orthotopically into the hippocampus of immunodificient nude mice (n=6). (A) *In vivo* bioluminescent imaging of nude mice at indicated time points. The right panel is the quantification of luminescence signal intensity. (B) Body weight of mice. All data are represented as the mean ± s.e.m. *p<0.05 (Student's t test). (C) Survival curve of mice. Medians are shown. *p<0.05 (Long-rank test).

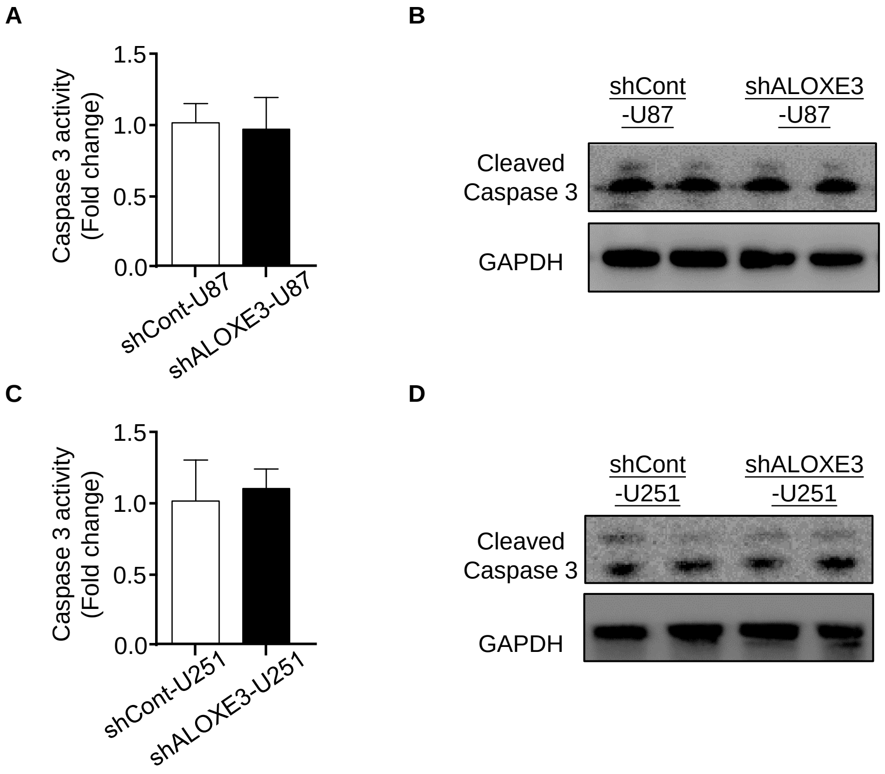

Figure S5. Apoptosis was not affected by ALOXE3 knockdown in GBM cells.
(A) Activity of caspase 3 in shALOXE3-U87 and shCont-U87 cells (n=6). (B) Immunoblotting analysis of Cleaved caspase 3 and GAPDH in shALOXE3-U87 and shCont-U87 cells. Representative immunoblot images are shown (n=6). (C) Activity of caspase 3 in shALOXE3-U251 and shCont-U251 cells (n=6). (D) Immunoblotting analysis of Cleaved caspase 3 and GAPDH in shALOXE3-U251 and shCont-U251 cells. Representative immunoblot images are shown (n=6). All data are represented as the mean ± s.e.m.

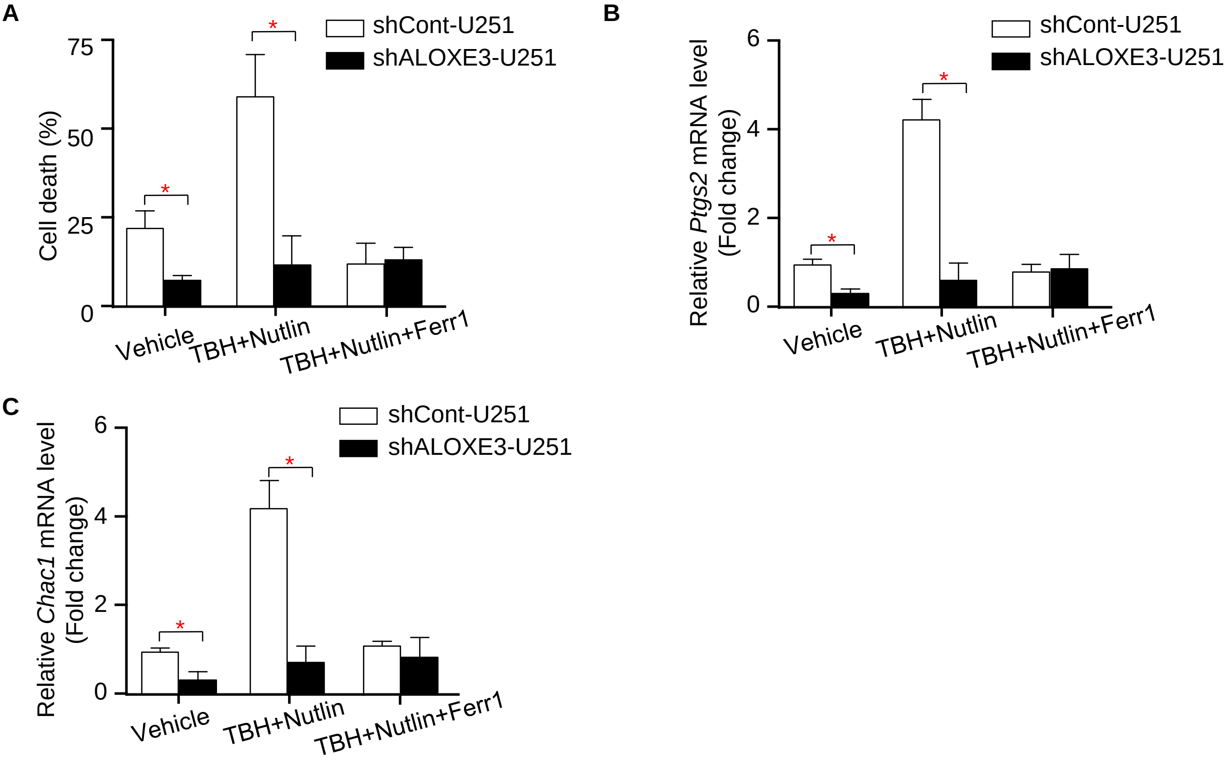

Figure S6. Resistance to ferroptosis was observed in U251 GBM cells with ALOXE3 deficiency. shALOXE3-U251 and shCont-U251 cells were pre-treated with Nutlin-3a for 12 hours and followed with TBH and Nutlin-3a +/- Ferr1 treatment for additional 8 hours (n=6). (A) Cells death. (B-C) Relative mRNA levels of (B) *Ptgs2* and (C) *Chac1* normalized with *GAPDH* in cells. All data are represented as the mean ± s.e.m. *p<0.05 (Student's t test).

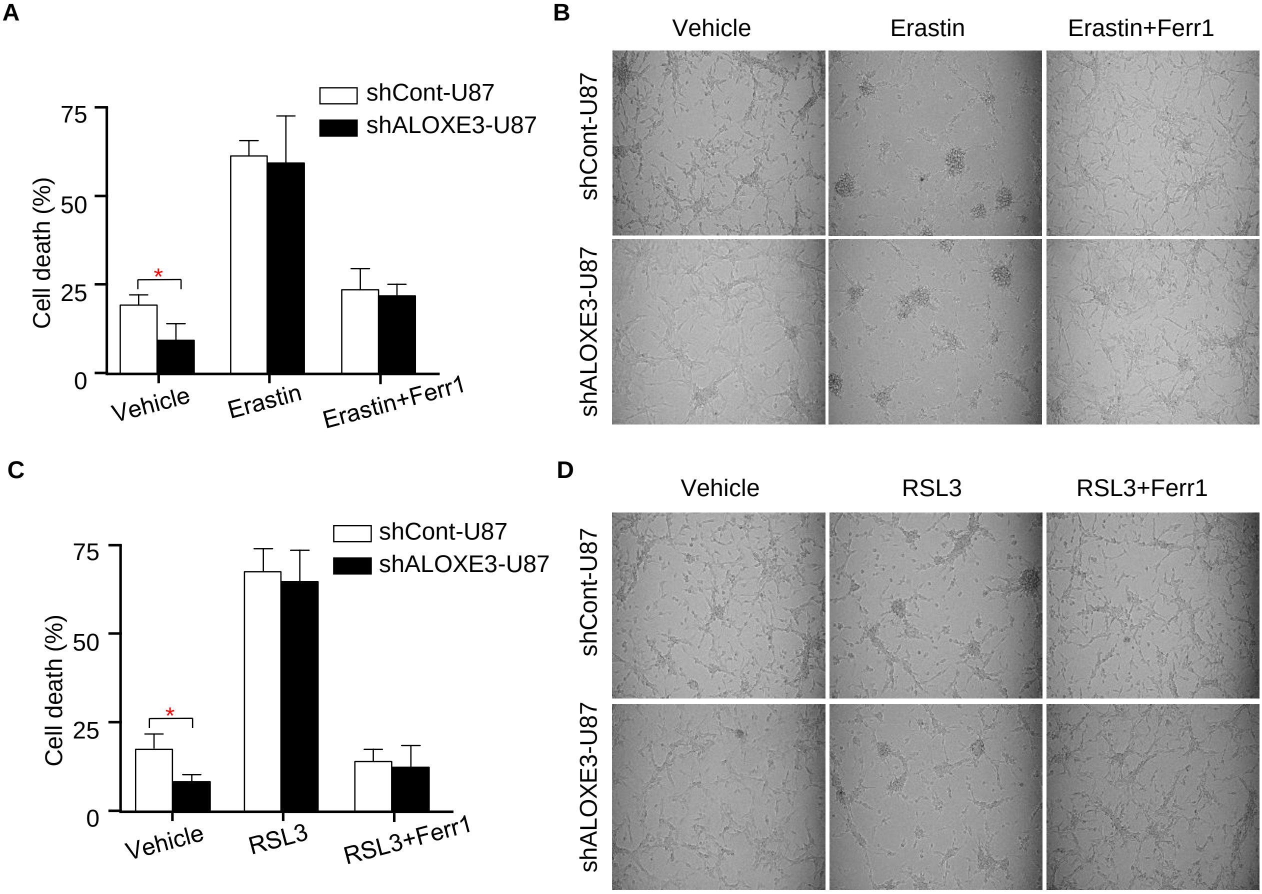

Figure S7. ALOXE3 is dispensable for ferroptosis induced by Erastin or RSL3.
(A-B) shALOXE3-U87 and shCont-U87 cells were treated with Erastin +/- Ferr1 for 12 hours (n=6). (A) Trypan blue staining for cells death. (B) Representative images of cells. (C-D) shALOXE3-U87 and shCont-U87 cells were treated with RSL3 +/- Ferr1 for 12 hours (n=6). (C) Trypan blue staining for cells death. (D) Representative images of cells. All data are represented as the mean ± s.e.m. *p<0.05 (Student's t test).

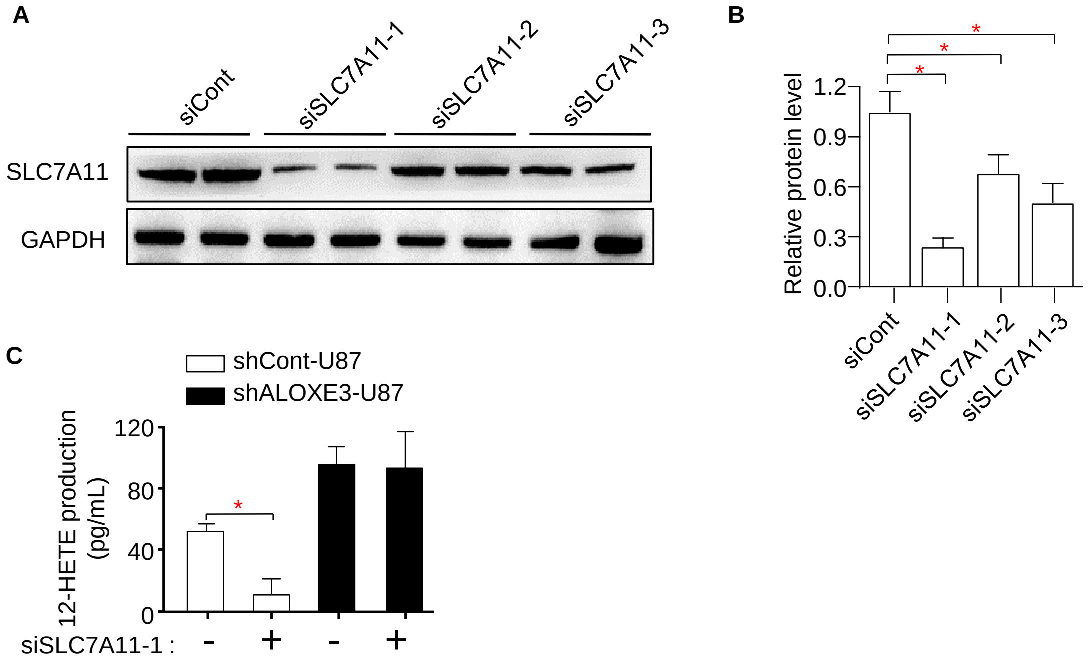

Figure S8. SLC7A11 silencing by siRNAs in U87 GBM cells.

(A) Immunoblotting analysis of SLC7A11 and GAPDH in U87 cells transfected with siRNAs against human SLC7A11. Representative images are shown. (B) The bar chart is relative expression level of SLC7A11 normalized with GAPDH (n=6). (C) 12-HETE levels of culture medium harvested from shALOXE3-U87 and shCont-U87 cells transfected with siSLC7A11-1 or siCont (n=6). All data are represented as the mean±s.e.m. *p<0.05 (Student's t test).

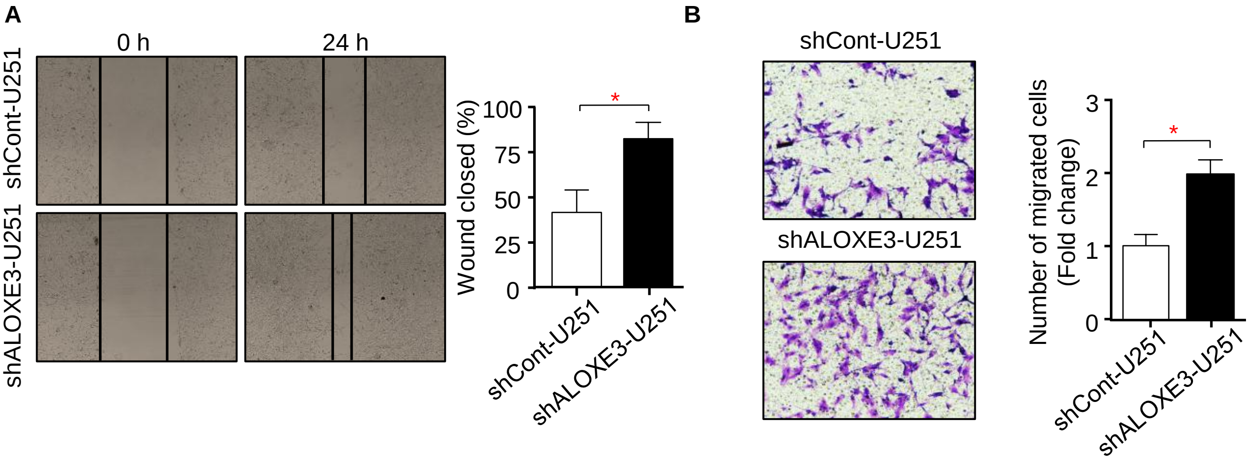

Figure S9. Enhanced migration in U251 GBM cells with ALOXE3 deficiency.
(A) Wound healing assay of shALOXE3-U251 and shCont-U251 cells was determined at 0 and 24 hours after wound was created. The right panel is the percentage of would closed at 24 hours (n=6). (B) Transwell assay of shALOXE3-U251 and shCont-U251 cells. The right panel is the quantification of the number of migrated cells (n=6). All data are represented as the mean ± s.e.m. *p<0.05 (Student's t test).

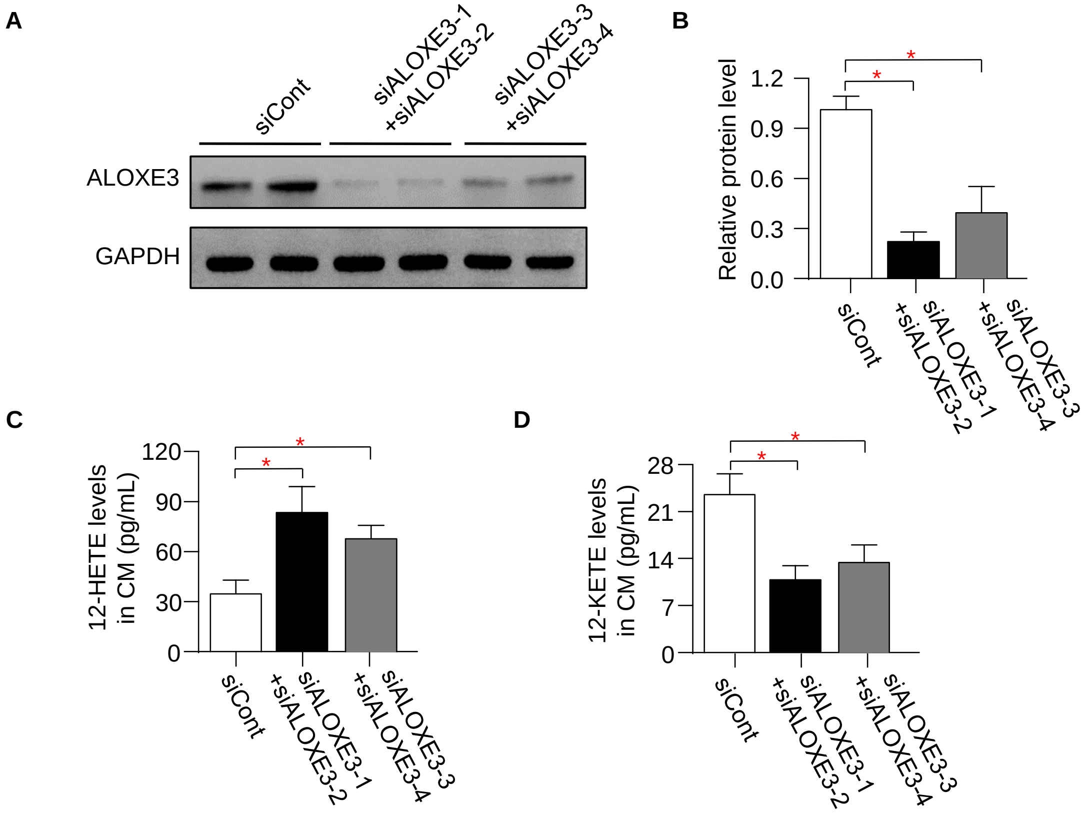

Figure S10. ALOXE3 silencing by siRNAs in U87 GBM cells.
(A) Immunoblotting analysis of ALOXE3 and GAPDH in U87 cells transfected with siRNAs against human ALOXE3. Representative images are shown. (B) The bar chart is relative expression level of ALOXE3 normalized with GAPDH (n=6). (C-D) Levels of (C) 12-HETE and (D) 12-KETE in culture medium harvested from U87 cells transfected with siALOXE3 or siCont (n=6). All data are represented as the mean±s.e.m. *p<0.05 (One-way ANOVA).

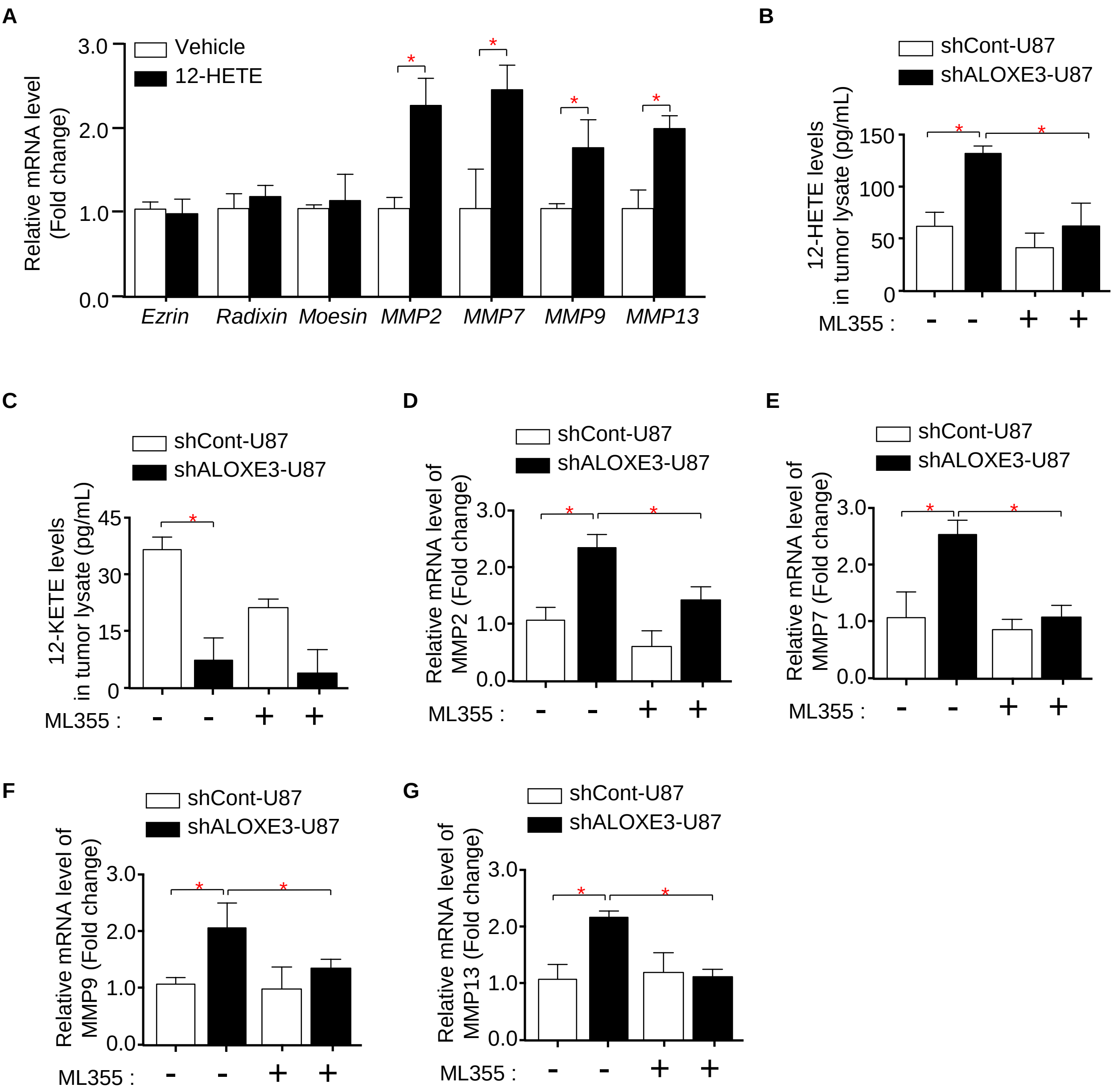

Figure S11. ML355 largely rescued the migration-promoting effects of shALOXE3-U87 cells in mice. (A) Relative mRNA levels of *Ezrin*, *Radixin*, *Moesin*, *MMP2*, *MMP7, MMP9* and *MMP13* normalized with *GAPDH* in cells treated with 12-HETE or Vehicle control were used (n=6). (B-G) shALOXE3-U87 and shCont-U87 cells were implanted orthotopically into the hippocampus of immunodificient nude mice, and followed by oral gavage with ML-355 (15mg/kg) or DMSO as vehicle control every two days (n=6). (B-C) Circulating levels of (B) 12-HETE and (C) 12-KETE in tumor lysates. (D-G) Relative mRNA levels of (D) *MMP2*, (E) *MMP7,* (F) *MMP9* and (G) *MMP13* normalized with *GAPDH.* All data are represented as the mean ± s.e.m. *p<0.05 (Student's t test).

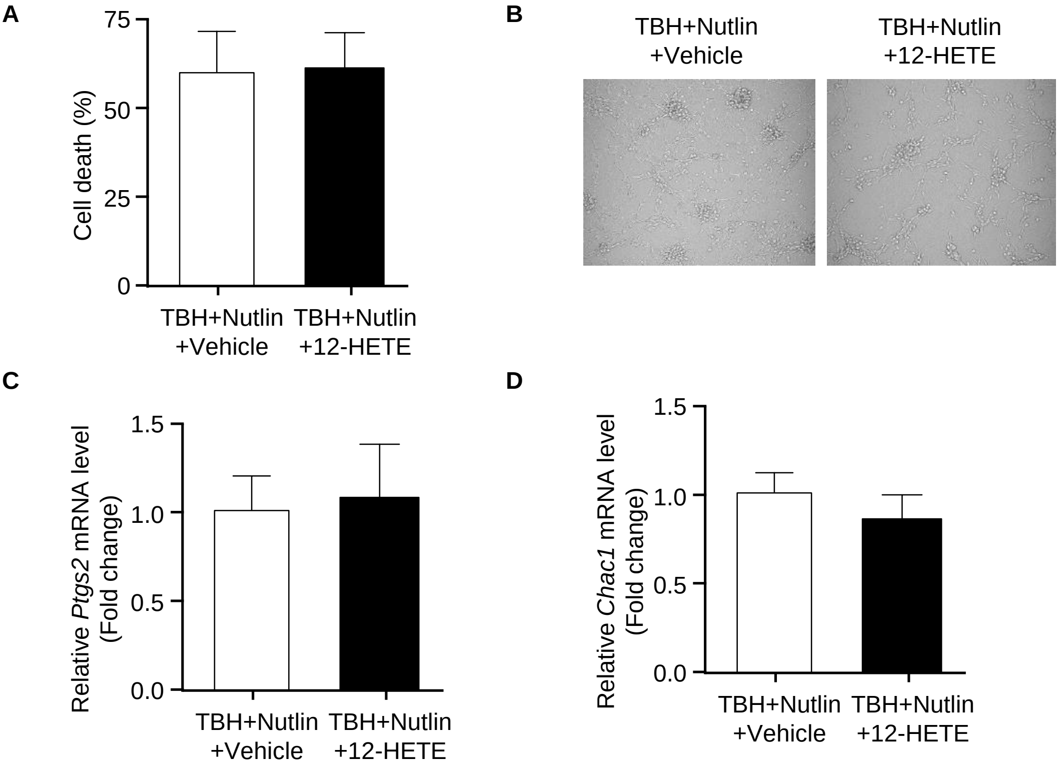

Figure S12. 12-HETE has no effect on p53-induced ferroptosis in GBM cells.
U87 cells with p53-induced ferroptosis (treated with TBH and Nutlin-3a) were subjected to 12-HETE or Vehicle treatment (n=6). (A) Trypan blue staining for cells death. (B) Representative images of cells. (C-D) Relative mRNA levels of (C) *Ptgs2* and (D) *Chac1* normalized with *GAPDH* in cells. All data are represented as the mean ± s.e.m. (Student's t test).

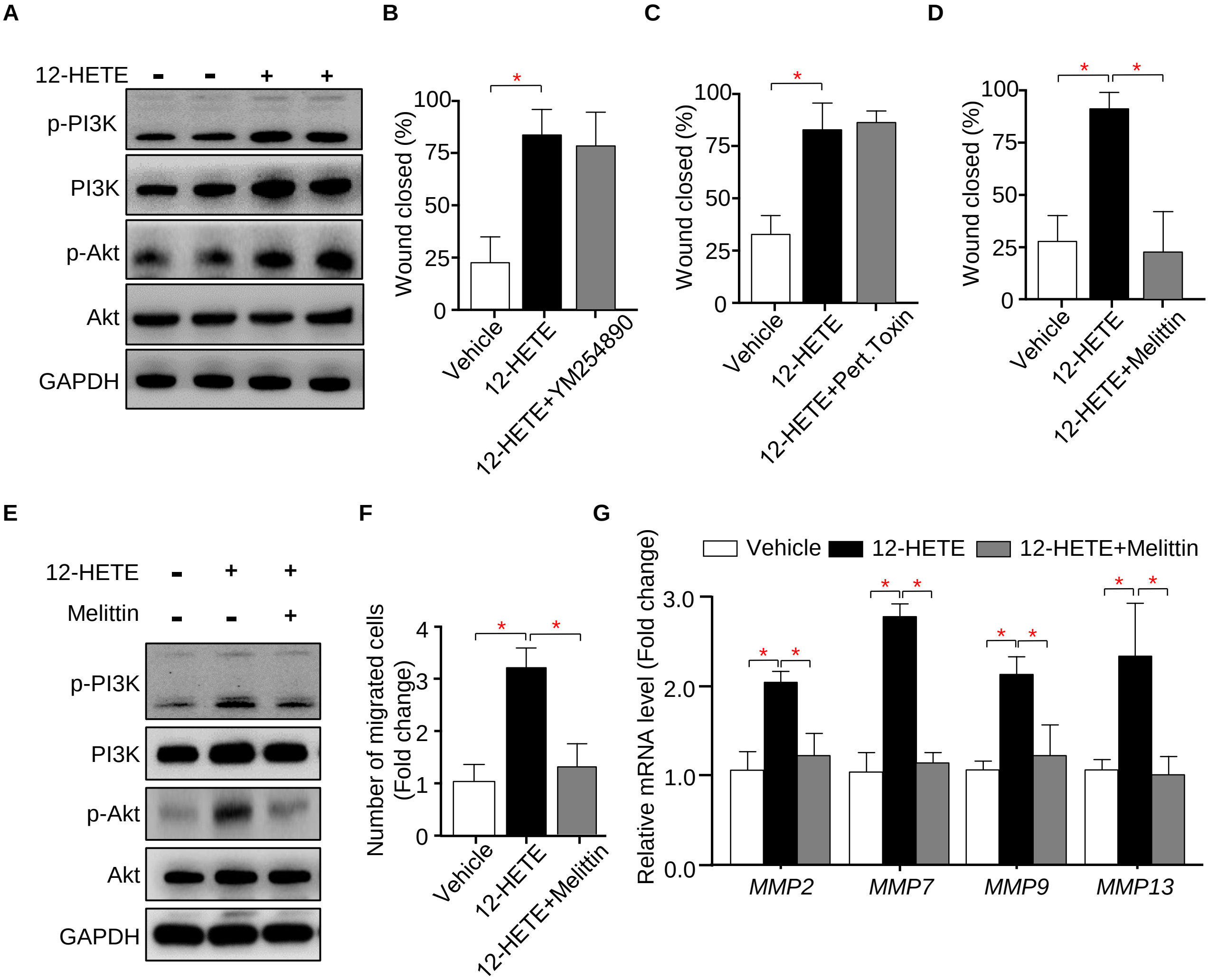

Figure S13. 12-HETE enhances migration of GBM cells via G_SPCR-PI3K-Akt pathway.
(A-D) U87 cells treated with 12-HETE or Vehicle control were used (n=6). (A) Immunoblotting analysis of p-PI3K, PI3K, p-Akt, Akt and GAPDH in cells. Representative immunoblot images are shown. (B-D) Wound healing assay of 12-HETE treated U87 cells pre-treated with (B) YM254890, (C) Pert.Toxin or (D) Melittin. (E-G) U87 cells treated with 12-HETE, 12-HETE + Melittin or Vehicle control were used (n=6). (E) Immunoblotting analysis of p-PI3K, PI3K, p-Akt, Akt and GAPDH in cells. Representative immunoblot images are shown. (F) Transwell assay. (G) Relative mRNA levels of *MMP2*, *MMP7, MMP9* and *MMP13* normalized with *GAPDH* in cells. All data are represented as the mean ± s.e.m. *p<0.05 (One-way ANOVA).

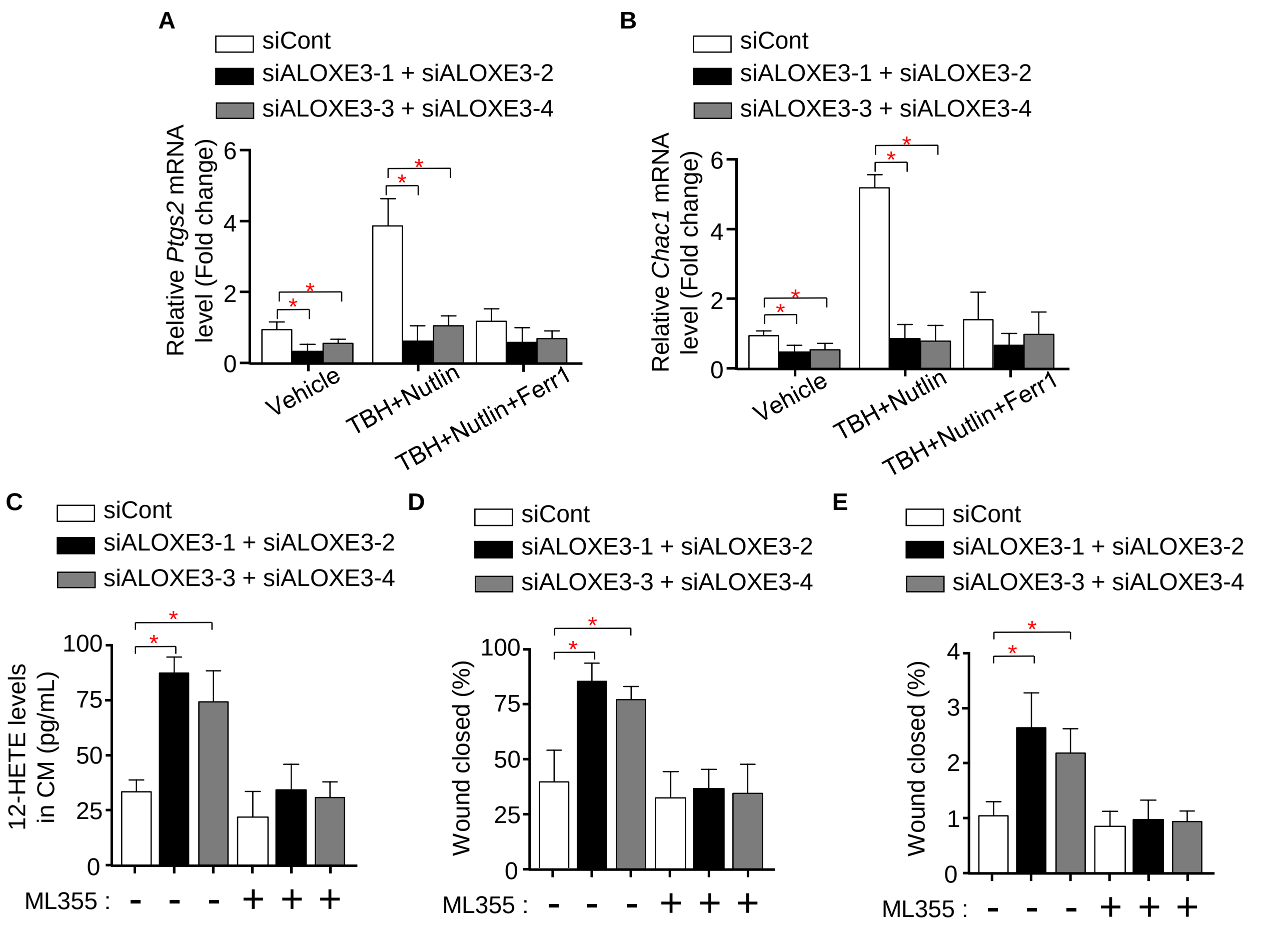

Figure S14. GBM-promoting functions of shALOXE3-U87 cells are not mediated by off-target effects. U87 GBM cells transfected with indicated siALOXE3 or siCont were used (n=6). (A-B) Cells were pre-treated with Nutlin-3a for 12 hours and followed with TBH and Nutlin-3a +/- Ferr1 treatment for additional 8 hours. Relative mRNA levels of (A) *Ptgs2* and (B) *Chac1* normalized with *GAPDH* in cells. (C-E) Cell were treated with ML355 or DMSO as control. (C) Circulating 12-HETE levels in culture medium. (D) Wound healing assay. (E) Transwell assay. All data are represented as the mean ± s.e.m. *p<0.05 (One-way ANOVA).

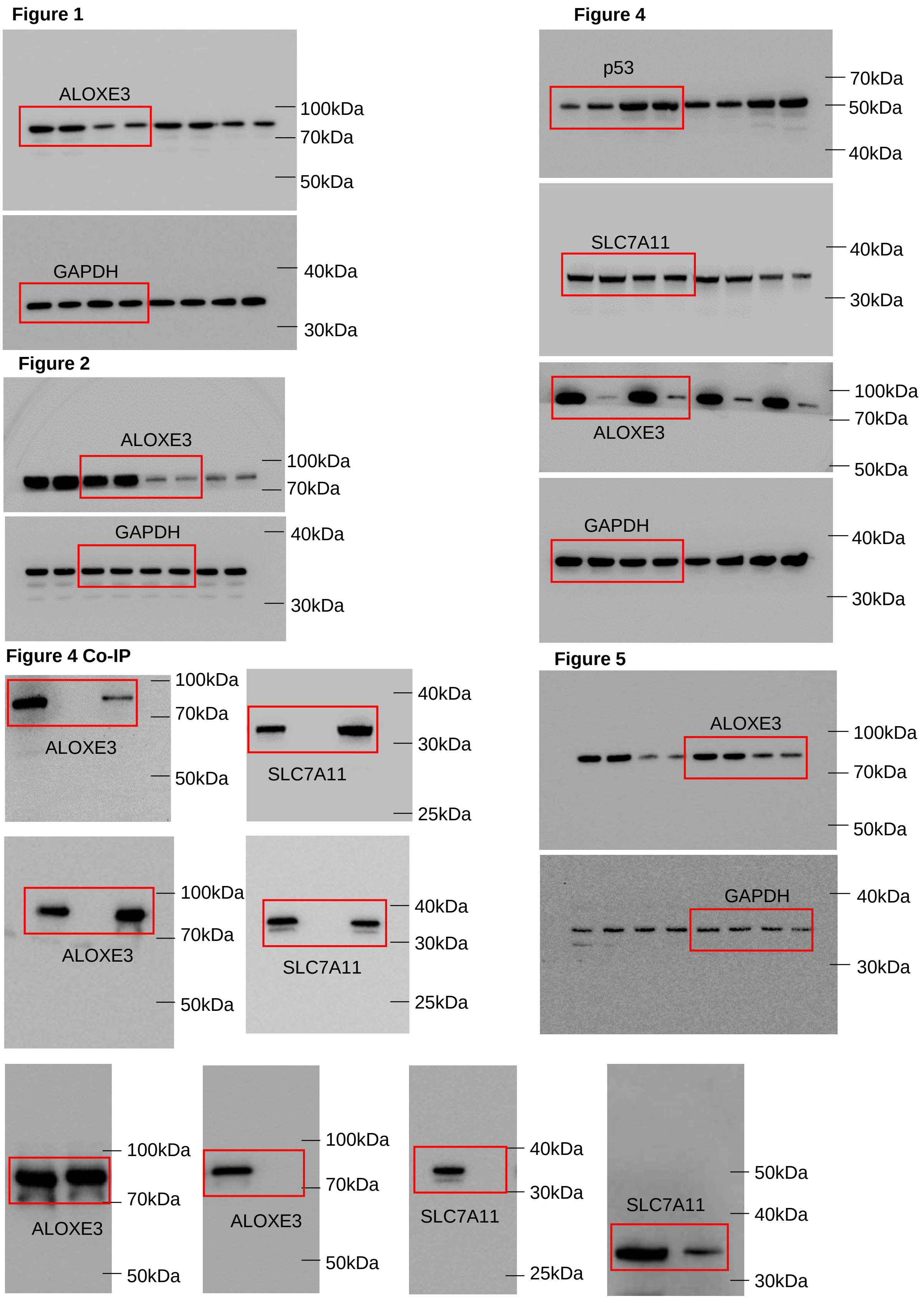

Figure S15. Full images of immunoblotting.

Supplementary Tables

Gene name	Sequences (5' to 3')	
ALOXE3	Forward	ACAACACGCACTTTCTGTGC
	Reverse	GGAGCTTGTAGATGGGGTGG
Ptgs2	Forward	CTTCACGCATCAGTTTTTCAAG
	Reverse	TCACCGTAAATATGATTTAAGTCCAC
Chac1	Forward	GAACCCTGGTTACCTGGGC
	Reverse	CGCAGCAAGTATTCAAGGTTGT
Ezrin	Forward	AGCACACGGAGCACTGCAGG
	Reverse	GTAACTCGGACATTGATTGG
Radixin	Forward	TATGCTGTCCAAGCCAAGTATG
	Reverse	CGCTGGGGTAGGAGTCTATCA
Moesin	Forward	TGTAAACCAGAGAGCTGCTGG
	Reverse	GAAGAGCACACATGAGACAGAGAA
MMP2	Forward	CAACTACAACTTCTTCCCTCGCA
	Reverse	GGTCACATCGCTCCAGACTTG
MMP7	Forward	GAGTGAGCTACAGTGGGAACA
	Reverse	CTATGACGCGGGAGTTTAACAT
MMP9	Forward	GCACCACCACAACATCACCTAT
	Reverse	AAACTGGATGACGATGTCTGCG
MMP13	Forward	TTGAGCTGGACTCATTGTCG
	Reverse	GGAGCCTCTCAGTCATGGAG
GAPDH	Forward	ATCAATGGAAATCCCATCACCA
	Reverse	GACTCCACGACGTACTCAGCG

Table S1. Primer sequences for mRNA expression analysis.

Primer name	Sequences (5' to 3')
miR-18a RT	GTCGTATCCAGTGCAGGGTCCGAGGTATTCGCACTGGATACGACCTATCT
miR-18a Forward	CCAAGGTAAGGTGCATCTAGTG
miR-18a Reverse	CAGTGCAGGGTCCGAGGTAT
U6 Forward	CTCGCTTCGGCAGCACATA
U6 Reverse	CGAATTTGCGTGTCATCCT

Table S2. Primer sequences for miRNA expression analysis.

Antibodies	Catalog Number
ALOXE3	#NBPI-32533; Novus
p53	#2524; Cell Signaling Technology
SLC7A11	#12691S; Cell Signaling Technology
PI3K	#4249; Cell Signaling Technology
p-PI3K	#4228; Cell Signaling Technology
Cleaved Caspase 3	#9664; Cell Signaling Technology
Akt	#ab179463; Abcam
p-Akt	#ab38449; Abcam
GAPDH	#2118; Cell Signaling Technology
Anti-mouse secondary antibody	#7076S; Cell Signaling Technology
Anti-rabbit secondary antibody	#7074S; Cell Signaling Technology

Table S3. Antibodies for immunoblotting.

shRNAs	Sequences (5' to 3')	
ALOXE3 shRNA1	Sense	CCGGACCAAAGTCCAATGCACAATACTCGAGTATTGTGCATTGGACTTTGGTTTTTG
	Antisense	AATTCAAAAAACCAAAGTCCAATGCACAATACTCGAGTATTGTGCATTGGACTTTGGT
ALOXE3 shRNA2	Sense	CCGGGCACTTTCTGTGCACGCATTTCTCGAGAAATGCGTGCACAGAAAGTGCTTTTTG
	Antisense	AATTCAAAAAGCACTTTCTGTGCACGCATTTCTCGAGAAATGCGTGCACAGAAAGTGC
ALOXE3 shRNA3	Sense	CCGGCCACTTCACCTACACCAATTTCTCGAGAAATTGGTGTAGGTGAAGTGGTTTTTG
	Antisense	AATTCAAAAACCACTTCACCTACACCAATTTCTCGAGAAATTGGTGTAGGTGAAGTGG
ALOXE3 shCont	Sense	CCGGGCTTCTCCGAACGTGTCACGTCTCGAGACGTGACACGTTCGGAGAAGCTTTTTG
	Antisense	AATTCAAAAAGCTTCTCCGAACGTGTCACGTCTCGAGACGTGACACGTTCGGAGAAGC

Table S4. Sequences of shRNAs.

siRNAs	Sequences (5’ to 3’)	
siSLC7A11-1	Sense	CAGAUAUGCAUCGUCCUUTT
	Antisense	AAGGACGAUGCAUAUCUGTT
siSLC7A11-2	Sense	GCAGCUACUGCUGUGAUATT
	Antisense	UAUCACAGCAGUAGCUGCTT
siSLC7A11-3	Sense	CCAUGAUUCAUGUCCGCATT
	Antisense	UGCGGACAUGAAUCAUGGTT
siALOXE3-1	Sense	UAAUACUUGUUUGCUUGCCTT
	Antisense	GGCAAGCAAACAAGUAUUATT
siALOXE3-2	Sense	AAGUUGUCGUCUUUGUCAATT
	Antisense	UUGACAAAGACGACAACUUTT
siALOXE3-3	Sense	GAUUUCUGAGACAAAGCUCTT
	Antisense	GAGCUUUGUCUCAGAAAUCTT
siALOXE3-4	Sense	UAGUAGCCCACGAUUUCUGTT
	Antisense	CAGAAAUCGUGGGCUACUATT
siCont	Sense	UUCUCCGAACGUGUCACGTT
	Antisense	CGUGACACGUUCGGAGAATT
miR-18a mimic	Sense	UAAGGUGCAUCUAGUGCAGAUAG
	Antisense	CUAUCUGCACUAGAUGCACCUUA
mimic-negative control	Sense	UUCUCCGAACGUGUCACGUTT
	Antisense	ACGUGACACGUUCGGAGAATT

Table S5. Sequences of siRNAs and miRNAs.
