# Supplementary material for: miR-18a promotes glioblastoma development by down-regulating ALOXE3-mediated ferroptotic and anti-migration activities
Source: Oncogenesis. 2021 Feb 12;10(2):15. doi: 10.1038/s41389-021-00304-3 (PMC7881152; doi:10.1038/s41389-021-00304-3)
Supplement: Supplementary file 2 — STR for U87 cells [file 41389_2021_304_MOESM2_ESM.pdf]

GENETIC TESTING BIOTECHNOLOGY

Cell Line Authentication Service
STR Profile Report

**Sample Submitted By:**	Dr. Zhuohao Liu<br>Shenzhen Hospital, Southern Medical University
**Email Address:**	lchouhoo@gmail.com
**Sales Order:**	200831B
**Cell Line Designation:**	U-87MG
**Date Sample Received:**	Aug 31th, 2020
**Report Date:**	Sep 4th, 2020

Methodology: Nineteen short tandem repeat (STR) loci plus the gender determining locus, Amelogenin, were amplified using the commercially available EX20 Kit from AGCU. The cell line sample was processed using the ABI Prism® 3130 XL Genetic Analyzer. Data were analyzed using GeneMapper® ID v3.2 software (Applied Biosystems). Appropriate positive and negative controls were run and confirmed for each sample submitted.

Data Interpretation: Cell lines were authenticated using Short Tandem Repeat (STR) analysis as described in 2012 in ANSI Standard (ASN-0002) by the ATCC Standards Development Organization (SDO) and in Capes-Davis et al., Match criteria for human cell line authentication: Where do we draw the line? Int J Cancer. 2013;132(11):2510-9.

GTB™ performs STR Profiling following ISO 9001:2008 and ISO/IEC 17025:2005 quality standards.

There are no warranties with respect to the services or results supplied, express or implied, including, without limitation, any implied warranty of merchantability or fitness for a particular purpose. Genetic Testing Biotechnology (GTB) is not liable for any damages or injuries resulting from receipt and/or improper, inappropriate, negligent or other wrongful use of the test results supplied, and/or from misidentification, misrepresentation, or lack of accuracy of those results. Your exclusive remedy against GTB and those supplying materials used in the services for any losses or damage of any kind whatsoever, whether in contract, tort, or otherwise, shall be, at GTB's option, refund of the fee paid for such service or repeat of the service.

NOTE: According to the recommendations of *IJC* on cell line authentication, the report is valid for 3 years since the issue date.

Technical Questions?
GTB Technical Support
+86-512-67486171
service@jsdna.org
Section 505, Yixin BLD
SIP, Suzhou, 215123
Jiangsu, P.R. China

Ordering Questions?
order@jsdna.org
GTB Corporation
+86-512-62806339
Section 303, Yixin BLD
SIP, Suzhou, 215123
Jiangsu, P.R. China

GENETIC TESTING BIOTECHNOLOGY

Cell Line Authentication Service
STR Profile Report

Sales Order: 200831B

	Test Results for Submitted Sample		ExPASy Reference Database Profile	
Loci	Query Profile: U-87MG		Database Profile: U-87MG ATCC	
Amelogenin	X		X	
D3S1358	16	17		
D13S317	8	11	8	11
D7S820	8	9	8	9
D16S539	12		12	
Penta E	7	14		
TPOX	8		8	
TH01	9.3		9.3	
D2S1338	20	23		
CSF1PO	10	11	10	11
Penta D	9	14		
D19S433	15	15.2		
vWA	15	17	15	17
D21S11	28	32.2		
D18S51	13			
D6S1043	11	18		
D8S1179	10	11		
D5S818	11	12	11	12
D12S391	18	21		
FGA	18	24		

The allele match algorithm compares the 8 core loci plus amelogenin only, even though alleles from all loci will be reported when available.

Note: Loci highlighted in grey (8 core STR loci plus Amelogenin) can be made public to verify cell identity. In order to protect the identity of the donor, ***please do not publish*** *the allele calls from all the STR loci tested. The sample match is based on the reference data available at the time of comparison.*

Explanation of Test Results

Cell lines with ≥80% match are considered to be related; i.e., derived from a common ancestry. Cell lines with between a 55% to 80% match require further profiling for authentication of relatedness.

☐ The submitted sample profile is human, but not a match for any profile in the ExPASy STR database.

☑ The submitted profile is an exact match for the following human cell line(s) in the ExPASy STR database (8 core loci plus Amelogenin): U-87MG ATCC

☐ The submitted profile is similar to the following ExPASy human cell line(s):

e-Signature, Technician: Digitally signed by Xuekun Chen
DN: cn=Xuekun Chen, o=Genetic Testing Biotechnology (Suzhou), ou=DNA Typing Section, email=order@jsdna.org, c=CN
Date: 2020.09.04 15:58:34 +08'00'

e-Signature, Reviewer: Digitally signed by Xiankun Zhao
DN: cn=Xiankun Zhao, o=Genetic Testing Biotechnology (Suzhou), ou=Supervision Section, email=service@jsdna.org, c=CN
Date: 2020.09.04 15:59:05 +08'00'

Addendum: Electropherogram for the customer's sample set 1 of 1

Cell Line Authentication Service
STR Profile Report

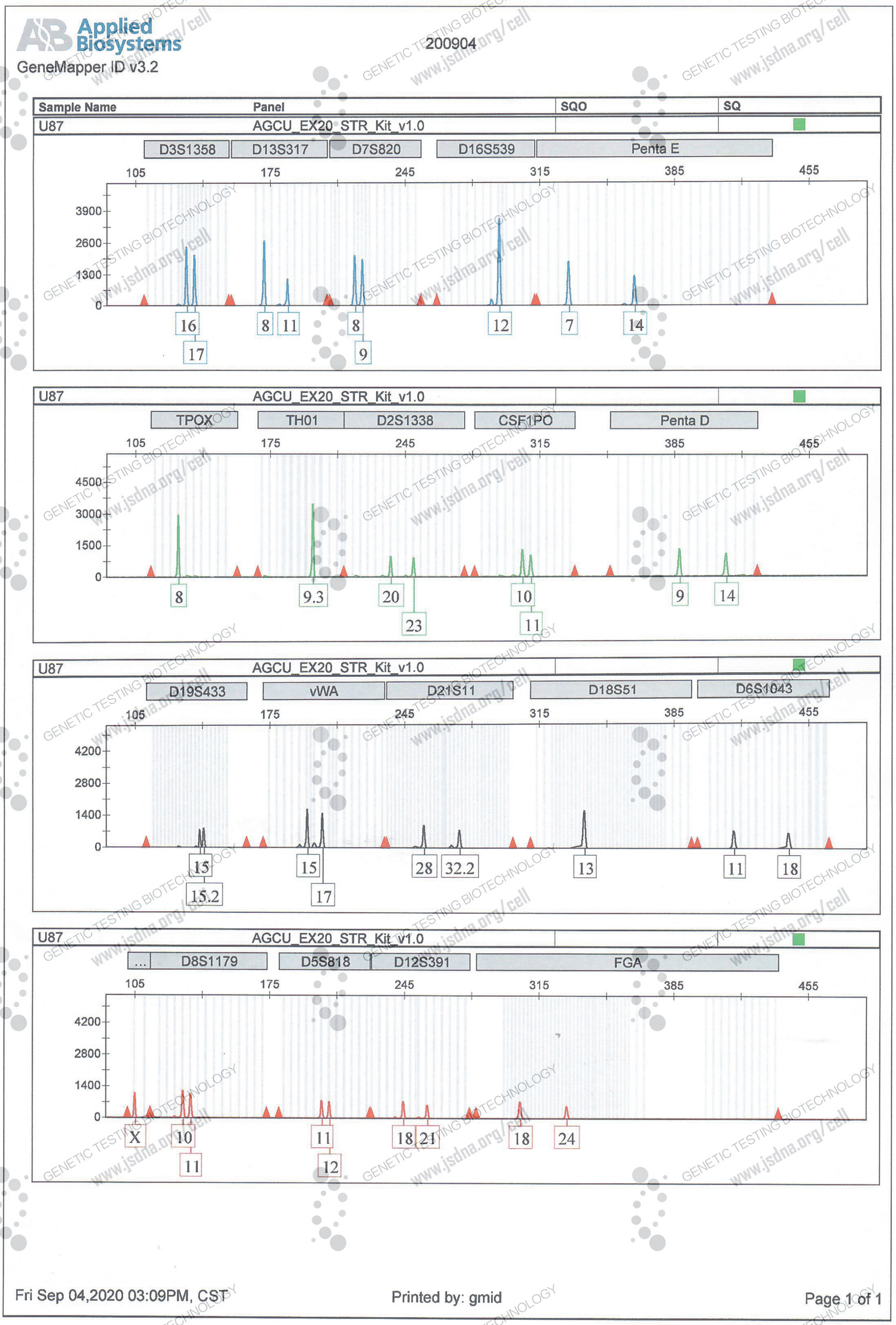

GENETIC TESTING BIOTECHNOLOGY www.isdna.org/cell

 Ver. 3.1.2
